# Genomic Insight into *Vibrio* Isolates from Fresh Raw Mussels and Ready-to-Eat Stuffed Mussels

**DOI:** 10.3390/pathogens14010052

**Published:** 2025-01-10

**Authors:** Artun Yibar, Muhammed Duman, Hilal Ay, Nihed Ajmi, Gorkem Tasci, Fatma Gurler, Sabire Guler, Danny Morick, Izzet Burcin Saticioglu

**Affiliations:** 1 Department of Food Hygiene and Technology, Faculty of Veterinary Medicine, Bursa Uludag University, Bursa 16059, Türkiye; fatmagurler34@gmail.com; 2 Department of Aquatic Animal Disease, Faculty of Veterinary Medicine, Bursa Uludag University, Bursa 16059, Türkiye; mduman@uludag.edu.tr (M.D.); nihed.ajmi.95@gmail.com (N.A.); grkmrocker12@gmail.com (G.T.); 3 Department of Molecular Biology and Genetics, Faculty of Arts and Science, Yildiz Technical University, Istanbul 34220, Türkiye; hilal.ay@yildiz.edu.tr; 4 Department of Histology and Embryology, Faculty of Veterinary Medicine, Bursa Uludag University, Bursa 16059, Türkiye; sabirepr@uludag.edu.tr; 5 Department of Blue Biotechnologies and Sustainable Mariculture, The Leon H. Charney School of Marine Sciences, University of Haifa, Haifa 3498838, Israel; dmorick@univ.haifa.ac.il

**Keywords:** whole-genome sequencing, foodborne pathogen, mussel, public health, *Vibrio* spp.

## Abstract

Consuming raw or undercooked mussels can lead to gastroenteritis and septicemia due to *Vibrio* contamination. This study analyzed the prevalence, density, species diversity, and molecular traits of *Vibrio* spp. in 48 fresh raw wild mussels (FRMs) and 48 ready-to-eat stuffed mussels (RTE-SMs) through genome analysis, assessing health risks. The results showed *Vibrio* prevalence rates of 12.5% in FRMs and 4.2% in RTE-SMs, with *V. alginolyticus* as the most common species (46.7%). It was determined that the seasonal distribution of *Vibrio* spp. prevalence in the samples was higher in the summer months. The genome sizes of the *Vibrio* spp. ranged from approximately 3.9 to 6.1 Mb, with the GC contents varying between 41.9% and 50.4%. A total of 22 virulence factor (VF) classes and up to six antimicrobial resistance (AMR) genes were detected in different *Vibrio* species. The presence of nine different biosynthetic gene clusters (BGCs), 27 prophage regions, and eight CRISPR/Cas systems in 15 *Vibrio* strains provides information about their potential pathogenicity, survival strategies, and adaptation to different habitats. Overall, this study provides a comprehensive understanding of the genomic diversity of *Vibrio* spp. isolated from FRM and RTE-SM samples, shedding light on the prevalence, pathogenicity, and toxicity mechanisms of *Vibrio*-induced gastroenteritis.

## 1. Introduction

Marine mussels, which are widespread in many areas in the world, have significant commercial value, and one of the most important mussel species is *Mytilus galloprovincialis* (the Mediterranean mussel), which also lives in the marine environments of Türkiye [1]. The amount of Mediterranean mussels produced in Türkiye in 2023 was reported as 2526.7 tons [2]. According to the World Bank’s 2023 data, 5136 kg of mussels was exported to 26 countries as live, fresh, and chilled [3], most of which were used for domestic consumption. Stuffed mussels, especially in Türkiye and other Mediterranean countries, are among the most consumed traditional foods, and they are usually sold by street stalls in Türkiye. In the production of stuffed mussels, shells of daily harvested fresh raw mussels *(Mytilus galloprovincialis*) are cleaned by scraping them with a knife. The shells are opened with a knife, and any beards and physical contaminants are removed and washed away. A pre-prepared mixture of pre-cooked rice, vegetable oil, salt, onions, herbs, and spices is stuffed manually into each shell, including mussel meat. Then, the shells are closed tightly before being cooked by means of steaming (to an internal temperature of ≥72 °C) [4,5].

Members of the genus *Vibrio*, commonly found in marine environments, are Gram-negative, rod-shaped, motile, facultative anaerobic pathogens [6,7,8,9]. The consumption of contaminated raw or undercooked seafood, especially bivalves, and exposure to contaminated water can cause vibriosis in humans [6,10]. *V. parahaemolyticus*, *V. cholerae*, *V. vulnificus*, *V. alginolyticus*, and *V. furnissii* are *Vibrio* spp. that have been identified as the causative agents of foodborne infections [9,11,12]. Some species that have not been reported to cause foodborne infections to date—such as *V. jasicida* (formerly named *V. harveyi*), *V. barjaei*, *V. rumoiensis*, *V. diabolicus*, and *V. owensii*—have significant implications for ecological, human, and animal health due to their widespread presence in marine environments and their potential to cause aquatic and zoonotic diseases [13,14,15,16,17]. They can threaten the food chain by infecting aquatic organisms and transferring pathogenic genes to other bacterial species [18]. From an ecological perspective, these species play a crucial role in nutrient cycling, organic matter decomposition, and maintaining microbial biodiversity in marine ecosystems. For instance, *V. barjaei* is a member of the Mediterranei clade, often found in association with marine bivalves such as clams, demonstrating its role in marine bivalve microbiomes [14]. Similarly, *V. rumoiensis*, known for its high catalase activity, thrives in oxidative environments such as wastewater from fishery processing plants, highlighting its potential for bioremediation and wastewater treatment applications [13]. Regarding animal health, *V. owensii* has been identified as a severe pathogen for aquaculture species, particularly the ornate spiny lobster (*Panulirus ornatus*), in which it causes larval mortality rates of up to 89% within 72 h post-infection. This infection is vectored by live feed organisms such as *Artemia*, further exacerbating its spread in aquaculture systems. The capacity of *V. owensii* to colonize the hepatopancreas and cause systemic infections underscores its threat to aquaculture sustainability and the necessity for effective control strategies [19]. In terms of human health, several *Vibrio* species have zoonotic potential. *V. diabolicus* has been linked to human infections, as isolates from human samples have been identified alongside environmental isolates, raising concerns about its pathogenic potential [17]. *V. jasicida* and *V. rotiferianus* have been isolated from shellfish in the United Kingdom for the first time, signaling the potential risk of seafood-borne infections, especially as warming sea-surface temperatures promote the growth and survival of *Vibrio* species. Additionally, *V. jasicida* is considered to be an emerging aquaculture pathogen, which could pose dual threats to aquatic animal health and human health through seafood consumption [20].

Marine mussels, as filter feeders, are continually exposed to a wide array of microorganisms, including potentially pathogenic bacteria that can threaten their health. Mussels have the ability to collect and concentrate bacteria from the marine environment [21]. The bacterial and chemical composition of mussels is affected by many factors, such as the characteristics of the geographical region where they are located, climate, temperature, depth, and environmental pollution [22]. Mussels are highly sensitive to water pollution and serve as key water quality indicators [23]. Like other bivalve mollusks, such as oysters, they are considered to be a common source of vibriosis, a foodborne illness [21].

Alongside external microbial exposure, mussels also maintain complex interactions with their internal microbiota. *Vibrio* species are naturally present within the microbiota of healthy mussels and oysters, accumulating within their tissues and body fluids, such as hemolymph. Although *Vibrio* species are part of the normal microbiota in these shellfish, many are pathogenic to other animals and humans. The US Centers for Disease Control and Prevention (CDC) lists the three most important species of *Vibrio* causing human infections in the United States of America (USA) as *V. parahemolyticus*, *V. fulnificus*, and *V. alginolyticus*. It is known that 80,000 cases of vibriosis occur each year in the USA, and 52,000 of these cases occur because of the consumption of contaminated food [24]. As a result of infection caused by the foodborne pathogen *Vibrio* spp., the pathogenesis table is characterized by septicemia and gastroenteritis [21]. Marine environments, which constitute the natural habitats of *Vibrio* spp., serve as the reservoirs of various genes associated with pathogenesis and antimicrobial resistance (AMR). It is also known that marine environments play a role in the transfer of bacterial species to humans through the food chain [25]. AMR genes in *Vibrio* spp. have been a significant public health problem for decades [26].

Considering the widespread consumption of mussels and mussel-derived food in the diet worldwide, determining the contamination of *Vibrio* spp. in foods through deep genomic analysis is important for public health. The aims of this study were (I) to determine the prevalence, species diversity, and genomic characteristics of *Vibrio* spp. in samples of fresh raw mussels and ready-to-eat stuffed mussels via whole-genome analysis, and (II) to determine potential virulence mechanisms and risk factors of *Vibrio* species contaminating mussels and mussel products.

## 2. Materials and Methods

### 2.1. Sampling

During the 12 months between June 2022 and May 2023, fresh raw wild mussels (FRMs) (n = 48) and ready-to-eat stuffed mussels (RTE-SMs) (n = 48) were obtained monthly from four companies harvesting and processing mussels from the Marmara Sea in Türkiye. The locations of harvesting for these companies were as follows: R-1: Balikesir (40°34′41.8″ N 27°35′37.8″ E), R-2: Mudanya (40°22′59.8″ N 28°52′45.3″ E), R-3: Gemlik (40°28′18.2″ N 28°54′28.3″ E), and R-4: Istanbul (40°59′31.1″ N 29°00′46.1″ E). While freshly and daily prepared RTE-SMs are sold in restaurants under a cold chain, the RTE-SMs used in this study were obtained on a day-to-day basis from street vendors exposed to open-air conditions around the second half of the day. All of the samples (each sample containing 25 mussels) were packed in sterile bags and transferred to the laboratory below 4 °C within three hours [27]. At each sampling time, the air temperature at each sales point (SP) and the seawater temperature in each region where the mussels were harvested were measured using a handheld probe (Model 85, YSI Inc., Yellow Springs, OH, USA) [28].

### 2.2. Isolation

For the isolation and selection of *Vibrio* spp., the inter-shell contents of each sample (n = 25) were mixed, and 10 g of the mixture from each sample was transferred to sterile stomacher bags (Seward Medical, London, UK). Then, 90 mL of sterile Maximum Recovery Diluent (MRD, Oxoid, Thermo Fisher, Milano, Italy) was added and homogenized with a stomacher (Seward™ Stomacher™ Model 400C Circulator Lab Blender, Fisher Scientific, Waltham, MA, USA). Subsequently, a loopful of each homogenate was streaked onto the four different media, i.e., thiosulfate citrate bile salt sucrose agar (TCBS; Oxoid, Basingstoke, UK), marine agar (MA, BD Difco, Franklin Lakes, NJ, USA), tryptic soy agar (TSA; Oxoid, Thermo Fisher, Madrid, Spain) supplemented with 2% NaCl [29], and *Pseudomonas* isolation agar (BD Diagnostic Systems, Heidelberg, Germany) [30]. After incubation at 30 °C for 72 h under aerobic conditions, potential *Vibrio* spp. isolates were selected. All the selected isolates were sub-cultured in tryptic soy broth (TSB, Oxoid, Thermo Fisher, Madrid, Spain) and on TSA at 30 °C for 72 h. All the isolates were then confirmed by means of standard biochemical tests and stored at −80 °C for further analysis.

### 2.3. MALDI-TOF MS Identification

The species of the selected isolates were first determined by means of MALDI-TOF MS (matrix-assisted laser desorption/ionization time-of-flight mass spectrometry; Bruker Scientific LLC, Billerica, MA, USA) in accordance with the manufacturer’s instructions. Briefly, isolates were streaked onto BHI agar plates and incubated at 30 °C overnight. A fresh single colony was picked with a 1 μL inoculation loop and transferred directly to the 96-well MALDI target plate in a thin layer, overlaid with 1 μL of HCCA matrix solution (α-cyano-4-hydroxycinnamic acid dissolved in 50% acetonitrile [ACN], 47.5% deionized [DI] water, and 2.5% trifluoroacetic acid [TFA]), and air-dried at room temperature [24]. The spectra were then acquired and compared using BioTyper 3.1 software (Bruker Daltonics, Bremen, Germany). According to the manufacturer’s interpretation criteria, identification scores of ≥2.0 were considered for reliable identification at the species level.

### 2.4. Genome Sequencing, Assembly, and Identification

The genomic DNA of the *Vibrio* isolates was extracted using a QIAamp DNA mini kit (Qiagen, Hilden, Germany) according to the manufacturer’s instructions. The amount and purity of the DNA in each sample were measured at 260 nm and 260/280 nm wavelengths with a spectrophotometer (Multiskan Go, Thermo, Waltham, MA, USA). For comprehensive genome-based analyses, the genomes of the *Vibrio* strains were sequenced using the Oxford Nanopore ligation sequencing kit (SQK-NBD114-24, Oxford Nanopore Technologies, Oxford, UK) on the PromethION platform. Sequencing libraries were loaded onto a PromethION Flow Cell and sequenced for 24 h using the P2 Solo sequencer (ONT) following the manufacturer’s instructions.

The ONT Fast5 files were converted to FASTQ files using the Guppy basecaller (v6.1.7) in high-accuracy (HAC) mode to ensure optimal read quality. The resulting FASTQ files were uploaded to the BV-BRC (Bacterial and Viral Bioinformatics Resource Center) online server (https://www.bv-brc.org/ (accessed on 13 October 2024)) for further processing. The BV-BRC server conducted normalization and quality filtering using BBNorm (v38.90) to balance the sequencing depth and reduce redundancy, thereby mitigating errors during assembly caused by over-represented reads. Genome assembly was conducted using the Flye assembler (v2.9.1-b1780) after normalization. The assembly was further refined through two rounds of polishing using Racon (v1.4.20) and alignment correction with Minimap2 (v2.17-r974) to improve the accuracy and continuity of the contigs. Contigs were filtered by length and coverage, with a minimum length of 1000 bases and a coverage threshold of 5×, resulting in 5 high-quality contigs. Assembly quality was assessed using Quast (v5.2.0), which provided key metrics such as N50, total contig length, genome completeness, and number of contigs. Species identification of the strains was achieved using the TYGS (Type Strain Genome Server, https://tygs.dsmz.de/ (accessed on 13 October 2024)) [31], and genome annotations were performed using the NCBI Prokaryotic Genome Automatic Annotation system, as well as the Rapid Annotations Using Subsystems Technology (RAST) server (https://rast.nmpdr.org/ (accessed on 13 October 2024)) using the RASTtk pipeline [32,33]. The final draft genome sequences were deposited in the NCBI GenBank database following the completion of genome assembly, annotation, and species identification, in order to facilitate accessibility for comparative genomic studies and ensure compliance with data sharing guidelines. Separate phylogenomic trees were constructed using the BV-BRC for each species using the genome sequences of *V. alginolyticus*, *V. diabolicus*, *V. jasicida*, *V. furnissii*, and *V. owensii*, selected based on diverse host, country, and year data available in GenBank. The BV-BRC bacterial genome tree pipeline followed the methodology outlined by the codon tree method, which utilizes single-copy protein-coding genes conserved across the selected genomes. Genome sequences were curated to ensure that high-quality and complete genomes were included in the analysis. The pipeline identified conserved single-copy genes across all the *Vibrio* genomes, which were then aligned using MUSCLE. The aligned sequences were concatenated into a supermatrix, enabling the phylogenomic signal from multiple genes to be considered simultaneously. A maximum-likelihood phylogenomic tree was constructed using IQ-TREE, with the best-fit model of sequence evolution selected using ModelFinder (v1.6.12). A bootstrap analysis with 1000 replicates was performed to assess the robustness of the tree. The resulting phylogenomic tree was visualized using FigTree (v1.4.4), and evolutionary relationships between pathogenic and non-pathogenic strains, as well as among different *Vibrio* species, were inferred based on the tree’s topology [34].

### 2.5. Genome Analysis

The analyses of antibiotic resistance genes and virulence genes were conducted using the Resistance Gene Identifier (RGI) integrated within the Comprehensive Antibiotic Resistance Database (https://card.mcmaster.ca/analyze/rgi (accessed on 13 October 2024)) and the Virulence Factor Database (VFDB) (http://www.mgc.ac.cn/cgi-bin/VFs/v5/main.cgi (accessed on 13 October 2024)), respectively [35,36]. Moreover, the antiSMASH server (https://antismash.secondarymetabolites.org/ (accessed on 13 October 2024)) [37] was employed to identify the bioactive secondary metabolite gene clusters. Additionally, prophages were predicted from the genomes using the PHASTEST (PHAge Search Tool with Enhanced Sequence Translation) web server (https://phastest.ca/ (accessed on 13 October 2024)). The intact, questionable, and incomplete prophage sequences were defined by the score values of >90, 70 to 90, and <70, respectively [38]. The CRISPRCasFinder (https://crisprcas.i2bc.paris-saclay.fr/CrisprCasFinder/Index (accessed on 13 October 2024)) server was employed to identify clustered regularly interspaced short palindromic repeat (CRISPR)/CRISPR-associated (*cas*) gene sequences. The pathogenic potential of the *Vibrio* strains was preliminarily assessed using PathogenFinder (https://cge.food.dtu.dk/services/PathogenFinder/; accessed on 13 October 2024). This tool provides genomic-based predictions but is limited in its capacity to determine definitive pathogenicity [39]. In addition, the prediction, clustering, and visualization of genomic islands—i.e., the clusters of genes of probable horizontal transfer origin—and associated antimicrobial resistance genes across the genomes were achieved using IslandCompare, available at https://islandcompare.ca/ (accessed on 13 October 2024) [40].

## 3. Results

The data highlight significant seasonal fluctuations in seawater temperatures, potentially influencing mussels’ biology and associated microbial communities. The summer months (June to August) exhibited the highest temperatures across all the regions, with R-1 recording a peak temperature of 34.8 °C in August, significantly higher than that of the other regions during the same period. Table 1 presents a comprehensive analysis of the seawater temperatures across the four different mussel-harvesting regions in the Marmara Sea, Türkiye, spanning a full year from June 2022 to May 2023.

Table 1 also highlights the air temperature variations at the SPs of the RTE-SM samples across the four regions over a year, from June 2022 to May 2023, in the Marmara Sea, Türkiye. Each SP exhibited unique air temperature trends. There was a clear pattern of seasonal temperature fluctuations, with peaks during summer and dips during winter (December to February). SP-1 had consistently higher temperatures throughout the year than other SPs, while SP-4 experienced significantly lower temperatures during winter, dropping to 2.6 °C in February.

Among the 96 samples (48 FRM and 48 RTE-SM), 6 (12.5%) of the 48 FRM samples and 2 (4.2%) of the 48 RTE-SM samples were contaminated with *Vibrio* spp. (Table 2). Based on the biochemical characteristics and colony type on isolation media and their sensitivity to a vibriostatic agent (O/129), the isolates were assumed to be *Vibrio* spp. A total of 15 *Vibrio* isolates belonging to seven species (*V. alginolyticus* [n = 7], *V. diabolicus* [n = 2], *V. rumoiensis* [n = 2], *V. jasicida* [n = 1, formerly known as *V. harveyi*], *V. furnissii* [n = 1], *V. barjai* [n = 1], and *V. owensii* [n = 1]) were identified in nine different samples. *V. alginolyticus* was the most prevalent species (46.7%), identified in the FRM and RTE-SM samples across various months, regions, and sales points. Although the genus-specific identification of *Vibrio* strains with MALDI-TOF MS was 93.3% (14/15), the species-specific identification was 66.7% (10/15), consistent with NGS. The results revealed that *Vibrio* spp. varied between the FRM and RTE-SM samples and across the regions in the Marmara Sea, Türkiye. A pattern emerged where higher seawater temperatures, especially in the summer months, were correlated with the increased detection of *Vibrio* spp. in the FRM and RTE-SM samples.

### 3.1. Sequence Analysis

The draft genome sequences of the *Vibrio* strains were obtained. The genome sizes of the strains ranged from approximately 3.9 to 6.1 Mb, with the GC contents varying between 41.9% and 50.4%. The general characteristics of the genomes and identification results are presented in Table 3.

### 3.2. Phylogenomics

The phylogenomic relationships of *V. alginolyticus*, *V. diabolicus*, *V. furnissii*, *V. owensii*, and *V. jasicida* were examined using publicly available genome data; totals of 37, 33, 31, 31, and 24 genomes, respectively, were included for these species, with all the genomes providing sufficient data for analysis except for one genome of *V. jasicida*, which was excluded due to incomplete information. The analysis revealed significant genomic diversity among the species. While *V. owensii*, *V. diabolicus*, and *V. jasicida* exhibited heterogeneous genome similarity with strains from the NCBI database, *V. alginolyticus* and *V. furnissii* were positioned in well-defined phylogroups, closely related to strains available in the database.

**Figure 1:**
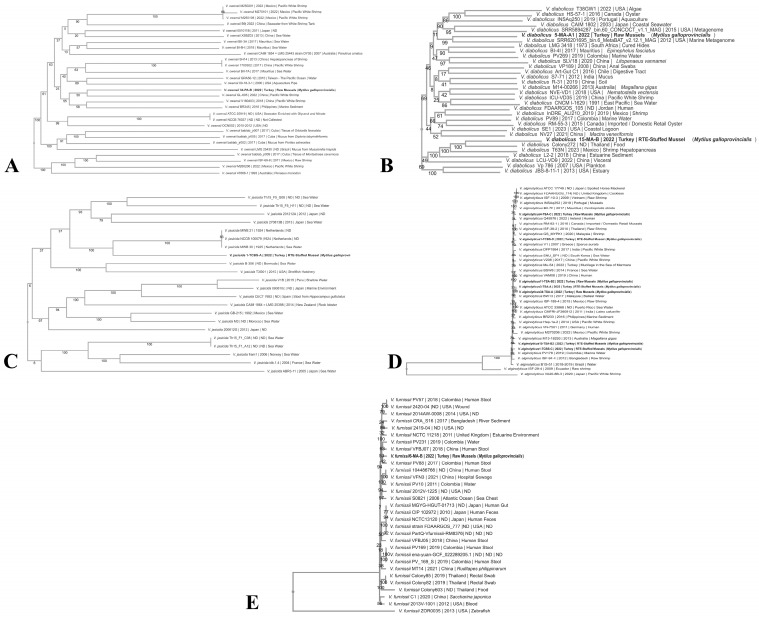
Phylogenetic tree of *V. owensii* 34-PA-B (**A**); *V. diabolicus* 5-MA-A1 and 15 MA-B (**B**); *V. jascida* 1-TCBS-A (**C**); *V. alginolyticus* 1-TCBS-C, 1-TCBS-D, 3 TSA-A, 4-TSA-C, 11-TSA-B2, 15-TSA-B2, and 34-TSA-A (**D**); and *V. furnissii* 6-MA-B (**E**) strains compared with the related *V. owensii*, *V. diabolicus*, *V. jascida*, *V. alginolyticus*, and *V. furnissii* strains documented in GenBank, collected from various samples in different years and geographical regions.

The phylogenetic analysis identified species-specific relationships, shedding light on the evolutionary connections of these strains. *V. owensii* strain 34-PA-B was clustered with strains previously reported from Pacific white shrimp and marine sediment in China and the Philippines (Figure 1A). *V. diabolicus* strains 5-MA-A1 and 15-MA-B were located in distinct phylogenetic clusters, closely related to strains from the marine metagenome of the USA and food samples from Thailand (Figure 1B). The *V. jasicida* strain was grouped near strains isolated from seawater in the Netherlands, suggesting a potential geographical link or shared environmental niche (Figure 1C). The seven strains of *V. alginolyticus* exhibited broad genomic diversity, forming distinct clusters with isolates from marine water, raw shrimp, Pacific white shrimp, humans, seawater, and mussels from regions such as Ireland, Malaysia, Mexico, Colombia, and Bangladesh (Figure 1D). This wide distribution highlights the adaptive potential of *V. alginolyticus* to diverse ecological niches. In contrast, *V. furnissii* strains demonstrated a closer genetic relationship with strains from human stool, hospital sewage, water, and estuarine environments in China, Colombia, the UK, and Bangladesh (Figure 1E), indicating potential public health significance.

These findings highlight the diverse evolutionary trajectories of *Vibrio* species, emphasizing the genetic links between the isolates from seafood, human-associated environments, and aquatic habitats. The clustering of strains from geographically distant regions suggests the potential for global dissemination, possibly facilitated by the movement of seafood products or the natural dispersal of marine organisms.

### 3.3. Biosynthetic Gene Clusters (BGCs) and Prophage

**Figure 2:**
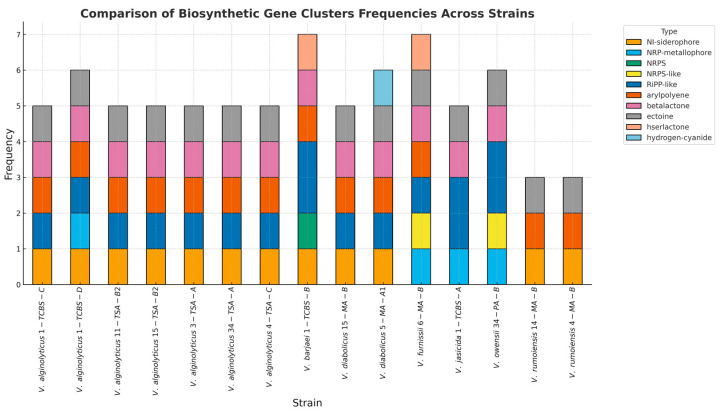
Biosynthetic gene clusters (BGCs) detected in the *Vibrio* genomes using the AntiSMASH server (https://antismash.secondarymetabolites.org/#!/start (accessed on 13 October 2024)).

A total of nine distinct biosynthetic gene cluster (BGC) types were identified in the *Vibrio* genomes, including ectoine, non-ribosomal peptide synthetase (NRPS)/NRPS-like fragment, non-ribosomal peptide metallophore (NRP-metallophore), aryl polyene (APE), beta-lactone, ribosomally synthesized and post-translationally modified peptide product (RiPP-like), NI-siderophores, homoserine lactones (hserlactones), and hydrogen cyanide (HCN). The number of BGCs varied across the 15 *Vibrio* strains, ranging from three to eight BGCs per strain, with *V. rumoiensis* 4-MA-B having the lowest count (three BGCs) and *V. furnissii* 6-MA-B having the highest (eight BGCs). On average, each strain contained approximately six BGCs. The analysis revealed that NI-siderophore clusters were present in 12 of the 15 strains, showing similarity rates of 62% and 87% with aerobactin and other gene clusters, and a 100% similarity to vibrioferrin and related gene clusters. These findings, as illustrated in Figure 2, highlight the diversity and functional potential of BGCs in *Vibrio* strains, suggesting their role in survival, virulence, and adaptation to environmental conditions.

Prophage sequences and viral elements embedded in *Vibrio* genomes, which may influence bacterial virulence, diversity, and evolution, are summarized in Table S1. The analysis identified 27 prophage regions with lengths ranging from 5.4 to 63.6 Kb, of which 18 regions were classified as intact. The highest number of phage regions (four regions) was observed in the *V. alginolyticus* 4-TSA-C strain, while only one phage region was detected in each of the seven strains. These findings highlight the potential role of prophage elements in shaping the genomic diversity and functional potential of *Vibrio* species.

### 3.4. Antimicrobial Resistance (AMR) and Virulence Factor (VF) Genes

**Figure 3:**
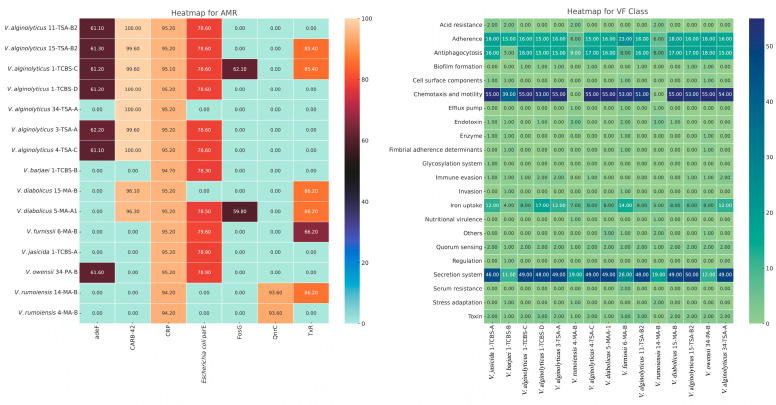
Combined heatmap of AMR and VF class data representing *Vibrio* spp. strain profiles.

Figure 3 and Table S2 show antimicrobial resistance (AMR) genes in the genomes of the *Vibrio* species isolated from the FRMs and RTE-SMs utilizing the Comprehensive Antibiotic Resistance Database (CARD). The cyclic AMP receptor protein gene (*CRP*), which controls multifactorial fluoroquinolone susceptibility [41], was detected in all the *Vibrio* strains, with homology levels ranging from 94.29% to 95.24%. Notably, a D476N single-nucleotide polymorphism (SNP) was found in the *Escherichia coli parE* gene in 73.3% of the strains, with homology values between 78.34% and 79.62%, suggesting a potential mechanism for quinolone resistance. The highest number of AMR genes was detected in the *V. alginolyticus* strains isolated from the RTE-SM samples, specifically in *V. alginolyticus* 1-TCBS-C (*TxR*, *adeF*, *FosG*, *bla_CARB-42_*, *CRP*, and *E. coli parE*) and *V. alginolyticus* 15-TSA-B2 (*adeF*, *CRP*, *TxR*, *qacG*, *bla_CARB-42_*, and *E. coli parE*). In contrast, the lowest number of AMR genes was observed in the *V. rumoiensis* 4-MA-B (*CRP* and *qnrC*) and *V. alginolyticus* 34-TSA-A (*bla_CARB-42_* and *CRP*) strains isolated from the FRM samples. The detection of *qnrC* in *V. rumoiensis* is significant, as it confers resistance to quinolones, a critical class of antibiotics. Resistance to tetracyclines was prevalent across the strains. The presence of the *TxR* and *adeF* genes, which are associated with tetracycline resistance, was observed in 10 strains. Additionally, the *FosG* gene, providing resistance to phosphonic acid derivatives, was identified in *V. alginolyticus* 1-TCBS-C and *V. diabolicus* 5-MA-A1. A noteworthy finding was the detection of the *qacG* gene, which encodes resistance to quaternary ammonium compounds (QACs), in *V. alginolyticus* 15-TSA-B2. This gene is linked to resistance against disinfectants and sanitizers, posing a significant concern for food processing environments. Beta-lactamase resistance genes, particularly *bla_CARB-42_* and *bla_CARB-56_*, were identified in several *Vibrio* strains. All the *V. alginolyticus* strains carried the *bla_CARB-42_* gene, with homology reaching 100% in some isolates, demonstrating resistance to penicillins and other β-lactam antibiotics. The highest prevalence of *bla_CARB-42_* was observed in the *V. alginolyticus* strains, whereas *bla_CARB-56_* was detected in *V. diabolicus* 5-MA-A1. The presence of these resistance genes indicates a strong capacity for resistance to β-lactams, which are crucial for clinical treatment. Overall, the presence of multiple AMR genes, including those encoding resistance to fluoroquinolones, tetracyclines, phosphonic acids, quaternary ammonium compounds, and β-lactams, highlights the multidrug resistance potential of these *Vibrio* species.

VF genes across 22 different VF classes, including adherence, anti-phagocytosis, chemotaxis and motility, enzyme, iron uptake, quorum sensing (QS), secretion system, toxin, acid resistance, biofilm formation, cell surface components, efflux pump, endotoxin, fimbrial adherence determinants, glycosylation system, immune evasion, invasion, nutritional virulence, others (O-antigen [*Yersinia*]), regulation, serum resistance, and stress adaptation, were detected, with notable differences in their presence across different *Vibrio* species (Table S3). Accessory colonization factors (*acfA*, *acfB*, *acfC*, and *acfD*) were not detected in our strains, although they were present in *V. cholerae* O1 biovar El Tor strain N16961 and *V. cholerae* O395. The QS molecule autoinducer-2 (AI-2; encoded by *luxS*) was detected in all the strains except for *V. barjaei* 1-TCBS-B. The other QS molecule, cholerae autoinducer-1 (CAI-1, encoded by *cqsA*), was not detected in two *V. rumeniensis* strains (4-MA-B and 14-MA-B) in this study. Our *Vibrio* strains were also closely related to those found in other bacterial taxa, such as *Escherichia*, *Aeromonas*, *Pseudomonas*, *Haemophilus*, *Yersinia*, *Burkholderia*, *Klebsiella*, *Streptococcus*, *Acinetobacter*, *Shigella*, *Coxiella*, *Helicobacter*, *Mycobacterium*, *Neisseria*, *Salmonella*, *Campylobacter*, and *Francisella*. The heatmap shows the distribution of VF gene counts across bacterial isolates. Rows correspond to the VF classes, while columns represent the bacterial isolates. The intensity of color indicates gene abundance, where darker red signifies higher counts, while blue represents lower counts. The hierarchical clustering of the bacterial isolates revealed distinct groupings, with isolates such as *V. diabolicus* and *V. alginolyticus* clustering together, while *V. rumoiensis* displayed a more distinct virulence profile (Figure 3).

### 3.5. Potential Human Pathogenicity

The pathogenic potential of the *Vibrio* strains was determined using the PathogenFinder tool (v1.1.) (Table 4 and Table S4). All the strains except for *V. barjaei* 1-TCBS-B and *V. rumoiensis* 4-MA-B were predicted as potential human pathogens. Based on these results, 13 *Vibrio* strains were predicted as potential human pathogens, with probabilities ranging from 0.535 to 0.864. *V. barjaei* 1-TCBS-B, from an RTE-SM sample, was categorized as a non-human pathogen, with a lower probability of 0.457. The highest probabilities of being human pathogens were attributed to the RTE-SM isolate *V. diabolicus* 15-MA-B (86.4%, with 49 pathogenic families) and the FRM strains *V. diabolicus* 5-MA-A1 (86.1%, with 49 pathogenic families), *V. alginolyticus* 11-TSA-B2 (84.9%, with 50 pathogenic families), and *V. alginolyticus* 4-TSA-C (84.9%, with 44 pathogenic families). The RAST annotation also confirmed that the genomes of the *Vibrio* strains encode genes for type I, II, III, IV, V, VI, and VII secretion systems, all of which play critical roles in VF delivery and interbacterial competition.

The analysis of CRISPR/Cas systems in *V. rumoiensis*, *V. diabolicus*, and *V. owensii* strains revealed the presence of CRISPR elements, but notably, no Cas (CRISPR-associated) genes were detected in any of the analyzed strains (Table S5). Only four *Vibrio* strains carried one or three CRISPR arrays, and in total, eight CRISPRs were identified, but no Cas genes. This finding is significant, as the functionality of the CRISPR system is typically associated with the presence of both CRISPR arrays and Cas genes, which together form the adaptive immune system of bacteria against phages and mobile genetic elements. CRISPR/Cas systems generally include a CRISPR array and Cas genes arranged in one or more operons. However, a significant proportion of CRISPR arrays are not adjacent to Cas genes [42], so we scrutinized the annotation of the genomes for the CRISPR-associated genes on the RAST tool.

The genome of *V. owensii* 34-PA-B has a CRISPR-associated protein of the Csx3 family with a 1224 bp length. The genome of *V. rumoiensis* 14-MA-B encodes a Csy3 family Cas protein (1026 bp), while the genome of *V. diabolicus* 5-MA-A1 also harbors a Csy4 family Cas protein (597 bp) adjacent to a Csy3 family protein (1044 bp). However, no CRISPR-associated proteins were detected in the genome of *V. diabolicus* 15-MA-B. One CRISPR array of 28 bp, consisting of two spacers, was identified in the genome of *V. rumoiensis* strain 4-MA-B. The genomes of *V. rumoiensis* 14-MA-B and *V. owensii* 34-PA-B showed multiple CRISPR arrays with varying spacer lengths and sequences, indicating active CRISPR systems potentially driven by diverse phage exposure. The CRISPR arrays found in the genomes of *V. diabolicus* 15-MA-B and *V. owensii* 34-PA-B had unique spacers, which are crucial for specifying the targets of the CRISPR defense mechanism. On the other hand, considering the low evidence level of CRISPR array detection in the CRISPRFinder tool and the absence of any Cas proteins, the result for *V. diabolicus* 15-MA-B might be evaluated as a false positive.

The CRISPR sequences identified in *V. rumoiensis*, *V. diabolicus*, and *V. owensii* displayed diverse consensus sequences. In *V. rumoiensis* 4-MA-B, two CRISPR elements were identified at positions 446,529–446,676 and 417,405–417,672, with consensus repeat sequences of “CTTACTAGCCACACACCCGATAGCACAC” at both loci. In *V. rumoiensis* 14-MA-B, the repeat sequences were “TTTCCTAGCTGCCTATTCGGCAGGTCAC” and “CGAGGTATTTTTGCAAACACGGA”, while in *V. diabolicus* 15-MA-B, the repeats were “TTTTGGAACAATAAAGTTTGTACAC” and “GTCATTCCGAGGAGCCTAAGCGA-CATCAGGAATCT”. Notably, *V. owensii* 34-PA-B had repeat sequences “GGCGGGTTCCGCGCTGGTTCCCGAGGCGGGGTCC” and “TTTAACCAAGATA-TAGGCCATTGGGATAC” at two distinct genomic loci. These diverse repeat sequences highlight the variability of CRISPR elements across *Vibrio* species.

Without Cas genes, the CRISPR arrays are unlikely to acquire new spacers, but the existing spacers may still play a role in bacterial regulatory processes. CRISPR arrays have been implicated in gene regulation, possibly acting as non-coding RNAs (ncRNAs) that influence transcription. In this context, CRISPRs may form secondary RNA structures that interact with regulatory proteins or small RNAs, thereby affecting gene expression.

**Figure 4:**
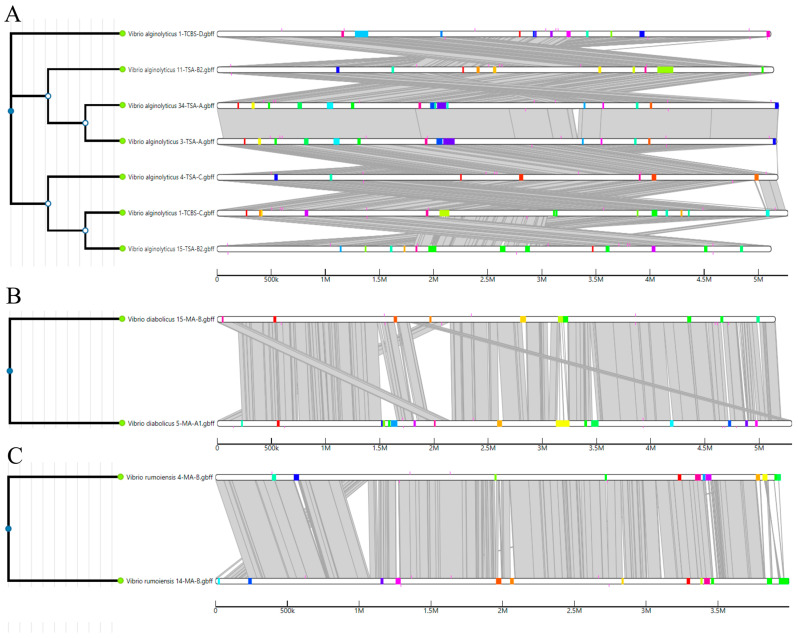
The genomic islands distributed in the genomes of the *Vibrio* strains isolated in the present study: (**A**) *V. alginolyticus* strains; (**B**) *V. diabolicus* strains; (**C**) *V. rumoiensis* strains. The phylogenetic tree on the left was calculated using Parsnp v1.2 based on single-nucleotide polymorphisms in the core genome of all the sequences submitted to the IslandCompare web server. The gray areas show the regions sharing sequence similarity across the pairs of aligned genomes. Genomic islands were colored with regard to sequence uniformity across the genomes.

Identifying genomic islands that encode mostly VFs, AMR genes, and other adaptation components is an effective way to use a population-based approach to genomic epidemiology and characterization [40]. The IslandCompare tool was used to detect and compare genomic islands found in the genomes of *V. alginolyticus*, *V. diabolicus*, and *V. rumoiensis* strains. Notably, *V. alginolyticus* strains showed considerable variation in the size, quantity, position, and identity of genomic islands encoded in their genomes (Figure 4 and Table S6).

## 4. Discussion

The main findings of this study were as follows: (i) Out of the 96 samples analyzed, 6 FRM samples (12.5%) and 2 RTE-SM samples (4.2%) were contaminated with *Vibrio* spp. (ii) A total of 15 *Vibrio* isolates from nine samples were identified, belonging to seven species. The most prevalent species was *V. alginolyticus*, which constituted 46.7% of the strains. We observed regional and seasonal variations in the prevalence of *Vibrio* spp., with a notable increase in contamination during the warmer summer months. (iii) Phylogenetic trees were constructed for five *Vibrio* species using genomic data from 33 to 37 genomes, depending on the species. The alignment of amino acids for phylogenetic analysis ranged from 252,823 to 379,159 amino acids. (iv) A genomic analysis identified nine different types of BGCs related to secondary metabolite production. The highest number of BGCs was eight, found in a *V. furnissii* strain. (v) Across the *Vibrio* isolates, a diverse array of AMR genes was detected. The *CRP* gene was found in all the strains, indicating a potential for broad-spectrum antimicrobial resistance. (vi) A prophage analysis within the *Vibrio* genomes revealed 27 prophage regions, 18 of which were intact. The presence of multiple VFs and secretion systems underscores the pathogenic potential of these strains.

Although *Vibrio parahaemolyticus* and *Vibrio cholerae* are well-known foodborne pathogenic *Vibrio* species, they were not detected in the FRM or RTE-SM samples in this study. In contrast to our findings, *V. parahaemolyticus* was reported in street food samples at a prevalence of 16.49% [43] and in fresh raw shellfish at a prevalence of 19.9% [44]. Additionally, contrary to our observations, *V. cholerae* was identified in FRM and RTE food samples in previous reports [45,46,47,48,49]. The most probable reason for the absence of *V. parahaemolyticus* and *V. cholerae* in our samples, despite their detection in previous studies, lies in the highly complex genetic similarity within the *Vibrio* genus. *V. cholerae* is monitored by the World Health Organization (WHO), and strict measures are taken to prevent the spread of cholera. The absence of *V. cholerae* could indicate that the mussels’ habitat was not contaminated by fecal matter or wastewater, which are common sources of this pathogen. This could reflect better water management or reduced anthropogenic impact in the sampling regions. The dominance of other *Vibrio* species, such as *V. alginolyticus*, in the sampled regions could also inhibit the growth and survival of *V. cholerae* due to competition for resources [50].

Our findings indicate a correlation between warmer months (June, July, and August) and an increased prevalence of *Vibrio* species, consistent with the elevated seawater and air temperatures during this period. Higher temperatures in the environment, particularly in seawater, seem to promote the growth and spread of *Vibrio* spp., as reported in previous studies [51,52]. For instance, in June, with seawater temperatures reaching 28.0 °C in R-1, we observed the presence of strains such as *V. alginolyticus* and *V. furnissii* in the FRM samples, supporting the idea that thermotolerant *Vibrio* species may contribute significantly to foodborne illnesses in summer [53]. *V. alginolyticus*, known for its resilience in warm conditions (up to 48 °C), was prevalent at multiple sampling points during these warmer months [54,55]. A study reported an 82.61% infection rate in sea bream during summer, which dropped to 30.23% in autumn [56]. Similarly, Mahmoud et al. (2022) [57] observed the highest incidence of *V. alginolyticus* in sea bass during summer (63.33%), followed by a much lower rate in winter (17.65%). This trend may be attributed to increased water temperatures during the summer, which create optimal conditions for the growth and proliferation of *V. alginolyticus*. Environmental factors, such as water quality and the presence of heavy metals, also play a critical role in the prevalence of *V. alginolyticus*. Elevated levels of heavy metals, including cadmium (Cd), lead (Pb), and nickel (Ni), have been linked to reduced fish immunity, making fish more susceptible to *Vibrio* infections [58,59]. In particular, *V. alginolyticus* has been found to have a positive correlation (*r* = 0.69) with higher water temperatures and increased heavy metal concentrations [60]. This suggests that elevated heavy metal concentrations may act as a stressor, weakening the immune systems of fish and providing *V. alginolyticus* with a competitive advantage over other microbial species. In addition, the concentration of iron (Fe) in the water has also been implicated in the higher prevalence of *V. alginolyticus*. Elevated iron levels recorded during the winter have been linked to increased *V. alginolyticus* infections. Iron is a key factor in bacterial growth and virulence, as it is an essential cofactor for many bacterial enzymes and metabolic pathways. As a result, increased iron availability may enhance the growth and colonization potential of *V. alginolyticus*, giving it an advantage over other species in the aquatic microbiota [61]. Collectively, the greater proportion of *V. alginolyticus* in our study may be attributed to a combination of seasonal temperature fluctuations, water quality parameters, and the bioavailability of heavy metals, including iron. These environmental factors create a favorable niche for *Vibrio* species. Furthermore, fish species, immunity status, and site-specific conditions likely contribute to the observed differences in the prevalence of *Vibrio* species between studies. Our findings are consistent with those of earlier reports highlighting the positive association between elevated temperatures, water quality, and *Vibrio* infections in aquaculture environments.

The consistent detection of *V. alginolyticus* and *V. diabolicus* in the RTE-SM samples from SP-3 in August, when temperatures were notably high (seawater at 25.4 °C and air peaking at 31.7 °C), suggests potential cross-contamination from raw mussels or inadequate cooking. Cross-contamination may occur through contact with raw mussel juices or improper cooking temperatures [62,63]. These findings underscore the importance of maintaining strict temperature controls during seafood preservation, keeping cold products below 5 °C and hot products above 60 °C, particularly in the summer months, to prevent *Vibrio* proliferation and ensure food safety [64,65,66].

Unlike previous studies that utilized sequence analyses at lengths of approximately 1.5–5 Kb, our study employed WGS with reads extending up to 6.1 Mb. Identifying *Vibrio* species at the genome level reduces the risk of the misidentification of closely related species, and it may also explain the different results between our study and previous studies. On the other hand, previous studies have determined that the genome lengths of *Vibrio* species have a wide range of 2.9–6.7 Mb, while their GC contents have a wide range of 38.0–57.2% [67]. The genome characteristics of the *Vibrio* species in our study are consistent with these studies, and these results also show that *Vibrio* genomes have high diversity. The phylogenetic analysis based on genome comparison showed that *V. owensii*, *V. diabolicus*, and *V. jasicida* are more heterogeneous than the *V. alginolyticus* and *V. furnissii* strains. While *V. owensii*, *V. diabolicus*, *V. jasicida*, and *V. alginolyticus* have been commonly found in mussels, marine water, and marine sediment, *V. funissii* is commonly reported from human stool, wound, and rectal swab samples in different countries. The genetic comparison shows a wide species distribution from different countries, from the USA to Thailand.

Several types of BGCs were identified in the *Vibrio* genomes, including those responsible for ectoine synthesis, NRPS/NRPS-like, APEs, beta-lactones, and NI-siderophores. These BGCs are recognized for their role in bacterial survival, virulence, biofilm formation, and utility in drug development [68,69,70,71,72,73]. All the *Vibrio* strains, except for *V. barjaei* 1-TCBS-B and *V. owensii* 34-PA-B, showed a significant presence of ectoine clusters, critical for the osmotic stress response in saline environments [74]. Notably, *V. alginolyticus* strains also exhibited a high incidence of siderophore-related clusters, enhancing their iron acquisition capabilities, which are vital in iron-depleted marine environments [75]. APE, a secondary metabolite thought to function in host immune system evasion, is widely found in Gram-negative pathogenic bacteria species [76]. APE BGCs were identified in all the strains except for *V. alginolyticus* 34-TSA-A, suggesting that they have protective properties against oxidative stress and are thought to play a role in biofilm formation [69]. Consistent with the findings in [77], the NRPS cluster detected in *V. jasicida* strain 1-TCBS-A in this study demonstrates the capacity of this strain to produce diverse and complex peptides that may have antibiotic properties. The antiSMASH analysis also detected homoserine lactone (hserlactone) clusters—which are generally used in the QS mechanism [78], which mainly regulates biofilm formation in microorganisms [79]—in the *V. barjaei* 1-TCBS-B and *V. furnissii* 6-MA-B strains.

Prophages can confer some features to their bacterial hosts, such as toxin production, resistance to environmental stresses, and enhanced virulence, which could impact the ecological fitness of *Vibrio* strains and their interactions with host organisms [80]. Certain prophages, such as PHAGE_Vibrio_VFJ_NC_021562 (in *V. alginolyticus* 4-TSA-C, *V. furnissii* 6-MA-B, and *V. alginolyticus* 11-TSA-B2) and PHAGE_Entero_DE3_NC_042057 (in *V. alginolyticus* 3-TSA-A and *V. alginolyticus* 34-TSA-A), were recurrent across multiple *V. alginolyticus* strains, suggesting a widespread distribution of these phage types within these *Vibrio* species. Consistent with our study [81], PHAGE_*Vibrio*_VFJ_NC_021562 was identified in many *V. parahaemolyticus* strains, but contrary to our results, they could not identify PHAGE_Entero_DE3_NC_042057, which had been previously identified in *E. coli* strains in only one study [82], in any *Vibrio* strain. These commonalities might reflect shared ecological niches or similar selective pressures. Identifying these prophages provides valuable insights into the genomic architecture and evolutionary dynamics of *Vibrio* species.

In light of the results that we obtained, numerous antimicrobial resistance mechanisms associated with *Vibrio* isolates were identified. One of the most prevalent mechanisms among pathogenic bacteria is the efflux pump system, which plays a significant role in both intrinsic and acquired antibiotic resistance in bacteria [83,84]; moreover, it is instrumental in regulating the internal environment by expelling toxic substances, quorum-sensing molecules, biofilm formation molecules, and bacterial VFs [85]. Another identified resistance mechanism was antibiotic target modification, which has become an increasingly common resistance strategy among pathogenic bacteria [86]. This resistance mechanism arises either as a result of genetic modifications within the bacteria or through enzymatic activities [86,87]. The resistance–nodulation–cell division (RND) efflux pump genes (*CRP* and *adeF*) were prevalent, suggesting a common resistance to multiple antibiotic classes, such as macrolides, fluoroquinolones, and penams [88]. In contrast to our findings, [89] reported that the *qnr* resistance gene, responsible for quinolone resistance, was the most common in 42 *Vibrio* strains isolated from shrimp. The presence of genes (*bla*_CARB-42_) responsible for β-lactamase resistance has been reported, especially in *V. alginolyticus* strains [90,91]. In the present study, as in other studies, the *bla*_CARB-42_ gene was detected in all the *V. alginolyticus* strains. The *V. alginolyticus* 15-TSA-B2 strain isolated from the RTE-SMs may have acquired resistance to disinfectants due to overuse/abuse and misuse in the RTE-SM production line [92]. *V. furnissii* strain 6-MA-B was also closely related to those found in other bacterial taxa, such as *Escherichia*, *Aeromonas*, *Pseudomonas*, *P. aeruginosa*, *Haemophilus*, *Klebsiella*, *Salmonella*, *Klebsiella*, *Salmonella*, *Burkholderia*, and *Yersinia*. Contrary to a study conducted in Italy [93], which showed the presence of aminoglycoside resistance genes (*aacC2* and *aadA*), a β-lactamase resistance gene (*bla_TEM_*), a quinolone resistance gene (*qnrS*), a tetracycline resistance gene (*tetD*), and a glycopeptide resistance gene (*vanB*) in 24–66% of the *Vibrio* strains isolated from mussels (*Mytilus galloprovincialis*), these genes were not detected in our *Vibrio* strains.

Compared to other studies conducted in different parts of the world, this study reveals some important similarities and differences related to the presence of virulence genes within *Vibrio* species isolated from various seafood samples. A study from Egypt reported that the collagenase gene was present in all strains of *V. alginolyticus* isolated from seafood. Similarly, the genomes of all the *Vibrio* strains in the present study, except for *V. barjaei* 1-TCBS-B and *V. furnissii* 6-MA-B, encoded microbial collagenase genes. In parallel to the previous study, no strain in either investigation transported the gene for thermostable direct hemolysin, *tdh* [94].

Similarly, a study from South China identified 11 virulence genes, such as *hflK*, *chiA*, and *flaC*, among the *Vibrio* strains isolated from marine fish samples. For the present work, we did not determine the presence of the *hflK* and *chiA* genes in any of our strains; however, the *flaC* gene was present in *V. barjaei* 1-TCBS-B and *V. furnissii* 6-MA-B [95]. In Malaysia, [96] reported the occurrence of the *chiA*, *luxR*, and *vhpA* genes at 66.7% in *Vibrio* strains isolated from marine fish. However, these genes were not detected in our strains. In a previous study, 98% of *Vibrio* strains were positive for the *tlh* gene coding for thermolabile hemolysin, while in our study, the corresponding value was 80%; while 68% of their strains contained the *flaC* gene, only 13.3% of our strains showed its presence. Agreement with that Malaysian study is seen in the absence of the genes *tdh* or *trh* in any of our strains. The gene *hlyA* was found only in the *V. furnissii* 6-MA-B strain; likewise, it was not found in their *Vibrio* strains. The *tox* gene was not detected in either study. In Japan [97], the genes *tlh*, *VPI*, *ompW*, *toxR*, and *ompU* were detected in *Vibrio* strains; none of these were identified in our strains. Similarly, although the *hlyA* gene was detected in 3.9% of their isolates, we detected it in only one strain (6.7%). Furthermore, the *tcpA*, *ctxA*, *ctxB*, and *tdh* genes were detected at 0% in their research, concurrent with the present study’s findings.

The accessory colonization factor, which is encoded by the *acfA*, *acfB*, *acfC*, and *acfD* genes, is required for efficient intestinal colonization and biofilm formation and found in *V. cholerae* O1 biovar El Tor strain N1696 and *V. cholerae* strain O395 [98]; it was not detected in any *Vibrio* strain in our study. The VFDB analysis also identified the presence of mannose-sensitive hemagglutinin (MSHA type IV pilus), which is crucial for DNA uptake for horizontal gene transfer [99], initial attachment, and the colonization of host cells [100]. This VF was detected in all the *Vibrio* species, highlighting its widespread distribution among pathogenic strains. None of the *Vibrio* strains obtained from the samples showed the presence of well-known toxin genes such as *ctxA* and *ctxB* (encoding cholera toxin), *vvhA* (encoding hemolysin/cytolysin), *tdh* (encoding thermostable direct hemolysin), or *rtxA*, *rtxB*, *rtxC*, and *rtxD* (encoding RTX toxin). However, the thermolabile hemolysin (encoded by *tlh*) was detected in all the strains except for a few—e.g., *V. barjaei* 1-TCBS-B, *V. rumoiensis* 4-MA-B, and 14-MA-B—indicating a common virulence mechanism that may contribute to the pathogenicity of these species in causing the lysis of red blood cells [101] and diarrheal diseases. Similarly to our study, TLH has been detected in *V. alginolyticus*, *V. diabolicus*, *V. harveyi*, and *V. parahaemolyticus* strains in previous studies [102,103,104]. Capsular polysaccharide (CPS) VF genes were found in all the strains, underscoring their role in evading host immune responses [105]. The CPS-related *wzc* gene was the most common among the strains (93.3%), but the CPS-associated *hp1* and *wbfT* genes were not detected in any isolates. Iron is critical for bacterial growth and virulence [106]. The widespread presence of the genes associated with iron uptake, such as enterobactin receptors (except in *V. jasicida* 1-TCBS-A, *V. barjaei* 1-TCBS-B, and *V. owensii* 34-PA-B) and heme receptors (except in *V. barjaei* 1-TCBS-B, *V. rumoiensis* 4-MA-B, and *V. rumoiensis* 14-MA-B), indicates that these *Vibrio* species have robust mechanisms for acquiring iron from the environment [107,108], which is essential for their survival and virulence. The absence of any flagella VF-associated genes in the *V. rumoiensis* 4-MA-B and 14-MA-B strains suggests possible differences in the motility and, hence, the infectivity of these strains. All the *Vibrio* genomes also contained factors of the type II secretion system. The widespread presence of genes encoding the extracellular protein secretion (EPS) type II secretion system across all the strains suggests a fundamental role for this system in the pathogenicity of *Vibrio* species, likely involved in the secretion of toxins and other effectors [109].

According to the results from PathogenFinder, potentially pathogenic *Vibrio* species are associated with different environmental niches [110] in the Marmara Sea of Türkiye. These predictions underscore the potential health risks posed by *Vibrio* strains in the FRM and RTE-SM samples, particularly those with high pathogenic potential, such as the *V. diabolicus* 5-MA-A1 and 15-MA-B strains. PathogenFinder served as a useful preliminary tool for estimating the pathogenic potential of the *Vibrio* strains based on genomic data. However, it should be emphasized that the tool has limitations, as it does not account for critical determinants of pathogenicity, such as VFs, sequence types (STs), serotypes, or host-specific traits. To address these limitations, complementary analyses, including the identification of VFs, AMR genes, and secretion systems, were performed in this study. These additional datasets strengthen the understanding of the potential pathogenicity of the studied *Vibrio* strains. Future research integrating genomic predictions with experimental validations will be necessary to fully assess their pathogenic risks.

Bacterial CRISPR/Cas systems, found in many bacteria, including *Vibrio* spp., provide adaptive immunity by defending against bacteriophages, plasmids, and other invaders [111]. Although CRISPR/Cas systems have been reported to be commonly found in *Vibrio* species [112,113,114], they were detected to a lesser extent in the present study. The results suggest that the four *Vibrio* strains (26.7%) possess a genetic defense mechanism against phage attack or invasion by foreign DNA—a crucial characteristic of these strains. Detecting CRISPR/Cas systems in the *Vibrio* species underscores the evolutionary arms race between these bacteria and their phage adversaries. The presence of these systems not only provides insights into the microbial immune mechanisms but also impacts the evolutionary dynamics of these bacteria, influencing their pathogenicity and survival strategies in marine environments and the food chain [114]. Previous studies [115,116] have demonstrated that “orphan” CRISPR arrays—those lacking Cas genes—can regulate gene expression through RNA interference-like mechanisms, such as base pairing with mRNA transcripts. Another explanation is that Cas genes may be located elsewhere in the genome or carried by mobile genetic elements, such as prophages or plasmids. Given that prophage sequences were detected in these strains, it is possible that the Cas genes required for the functionality of the CRISPR system are encoded within the phage-related regions. In some cases, the CRISPR system is co-opted by phages to serve as a defense mechanism against other phages, creating a phenomenon known as “CRISPR–phage warfare”. Therefore, the absence of Cas genes within the immediate vicinity of CRISPR arrays does not necessarily imply that the CRISPR system is non-functional. While no Cas genes were identified in the *V. rumoiensis*, *V. diabolicus*, or *V. owensii* strains, the detection of CRISPR arrays suggests that these elements may play regulatory roles or act as residual components of a previously active CRISPR/Cas system.

In gastroenteritis cases caused by *Vibrio* species, various VFs have been identified, including genes that encode toxins and other pathogenic mechanisms. While previous studies have often focused on thermostable direct hemolysin [117], our research has detected a range of virulence-associated genes, such as *tlh*, *V. cholerae* cytolysin, aerolysin (*AerA*), cytotoxic enterotoxin (*Act*), and the phytotoxins coronatine and phaseolotoxin. This finding underlines the diverse and complex mechanisms through which *Vibrio* species can cause disease. Additionally, non-O1/non-O139 *V. cholerae* serogroups exhibit T3SS-dependent virulence, which is essential for intestinal colonization and the onset of diarrhea. Documented outbreaks linked to these strains—such as those from the Chester River, USA—feature a composite genotype consisting of several virulence genes, including *hlyA*, *stn*, *sto*, *hap*, *rtxA*, *nanH*, *vcsC*, *vspD*, *vcsN*, *vcsV*, *vasA*, *vasK*, and *vasH*. The presence of these genes in environmental isolates underscores their potential to cause significant illness, revealing the adaptability of *Vibrio* strains in acquiring VFs [118]. The present study further highlights this complexity, as we detected many of these virulence genes in the non-O1/non-O139 *V. cholerae* isolates from mussel samples. The mechanisms through which *Vibrio* species can cause gastroenteritis involve a complex interplay of enzymes, toxins, and secretion systems. The discovery of a “virulence cocktail” in mussels suggests that consuming a single mussel harboring multiple *Vibrio* species with diverse virulence genes could lead to complex gastroenteritis symptoms and, potentially, to other severe health conditions. This underscores the importance of understanding and monitoring the full spectrum of VFs present in seafood in order to better assess public health risks.

## 5. Conclusions

The findings of this study provide valuable insights for identifying the genetic diversity and population structure of *Vibrio* spp. isolated from the FRM and RTE-SM samples. Various possible pathogenic *Vibrio* spp. were present in mussel-derived foodstuffs in this study, and most showed a detectable seasonal trend that makes these bacteria a potential food safety concern. The frequent detection of *V. alginolyticus* in several samples, especially during the warmer seasons, might suggest that higher environmental temperatures may increase the prevalence of diseases caused by pathogenic *Vibrio* species. Numerous strains, especially *V. alginolyticus*, carried VFs and AMR genes, indicating public health implications for seafood consumption. The analyses of BGCs, prophages, and the CRISPR/Cas system facilitated an extrapolation of the survival methods and the pathogenic capabilities of *Vibrio* strains. This study provides a foundation for future research, contributing valuable data on the ecological and genetic features of *Vibrio* spp., with significant implications for public health and marine microbiology.

To avoid gastrointestinal diseases from raw or RTE mussel products contaminated with *Vibrio* spp. and other seaborne pathogens, measures should include enforcing strict seawater quality standards, applying depuration processes, educating consumers and food handlers about the risks, promoting proper cooking methods, ensuring hygiene during handling and processing, maintaining proper service and sales conditions (particularly cold chain management), and increasing research on the AMR genes and VFs of these pathogens. These actions can significantly reduce health risks and improve public health outcomes.

## Figures and Tables

**Table 1:** The seawater and air temperature (°C) of each mussel-harvesting region (R) and sales point (SP) of ready-to-eat stuffed mussels in the Marmara Sea, Türkiye.

Region	June	July	August	September	October	November	December	January	February	March	April	May
R-1	28.0	22.3	34.8	21.1	20.0	16.0	8.7	11.5	9.2	10.1	11.4	15.8
SP-1	24.1	31.6	31.1	20.2	22.0	20.2	13.1	13.3	17.4	15.2	11.1	18.0
R-2	20.8	23.7	24.9	20.5	18.0	14.5	12.1	11.2	8.8	10.6	13.2	15.4
SP-2	21.8	25.3	29.8	19.9	23.0	23.0	14.2	9.1	17.6	14.6	12.7	19.1
R-3	21.0	23.6	25.4	21.3	17.6	15.5	13.7	10.8	9.3	9.2	11.2	13.9
SP-3	23.0	23.1	31.7	20.8	21.5	24.4	9.0	8.7	15.7	11.6	11.0	13.2
R-4	20.6	23.1	26.2	21.7	20.6	17.0	12.0	10.9	8.4	10.1	12.2	17.5
SP-4	23.2	25.0	29.7	19.3	21.0	20.0	8.0	11.0	2.6	9.9	11.1	19.8

R: region; SP: sales point.

**Table 2:** Identified *Vibrio* spp. isolates in fresh raw mussel (FRM) and ready-to-eat stuffed mussel (RTE-SM) samples.

Strain Name	Sample Type	Region/Sales Point	Sampling Month	MALDI-TOF MS Results	Genome-Based Phylogeny Results
1-TCBS-A	RTE-SM	SP-2	June 2022	*V. harveyi*	*V. jasicida*
1-TCBS-B	RTE-SM	SP-2	June 2022	NI	*V. barjaei*
1-TCBS-C	RTE-SM	SP-2	June 2022	*V. alginolyticus*	*V. alginolyticus*
1-TCBS-D	RTE-SM	SP-2	June 2022	*V. alginolyticus*	*V. alginolyticus*
3-TSA-A	RTE-SM	SP-3	June 2022	*V. rumoiensis*	*V. alginolyticus*
4-MA-B	FRM	R-3	June 2022	*V. rumoiensis*	*V. rumoiensis*
4-TSA-C	FRM	R-3	June 2022	*V. alginolyticus*	*V. alginolyticus*
5-MA-A1	FRM	R-4	June 2022	*V. alginolyticus*	*V. diabolicus*
6-MA-B	FRM	R-1	June 2022	*V. furnissii*	*V. furnissii*
11-TSA-B2	FRM	R-1	July 2022	*V. alginolyticus*	*V. alginolyticus*
14-MA-B	FRM	R-2	July 2022	*V. rumoiensis*	*V. rumoiensis*
15-MA-B	RTE-SM	SP-3	August 2022	*V. alginolyticus*	*V. diabolicus*
15-TSA-B2	RTE-SM	SP-3	August 2022	*V. alginolyticus*	*V. alginolyticus*
34-PA-B	FRM	R-1	October 2022	*V. harveyi*	*V. owensii*
34-TSA-A	FRM	R-1	October 2022	*V. alginolyticus*	*V. alginolyticus*

Note: The presence of TCBS in the strain names indicates that the isolate was isolated from TCBS medium, PA from Pseudomonas agar, TSA from tryptic soy agar, and MA from marine agar. RTE-SM: ready-to-eat stuffed mussel; FRM: fresh raw mussel; R: region; SP: sales point; NI: not identified.

**Table 3:** Genomic characteristics of the *Vibrio* strains isolated from fresh raw mussel (FRM) and ready-to-eat stuffed mussel (RTE-SM) samples.

Strains	GeneBank ID	Genome Size (bp)	Genome Coverage	No. Contigs	GC Content (%)	Total Genes	Protein-Coding Genes (CDSs)	rRNAs (5S, 16S, 23S)	tRNAs	ncRNAs	Pseudogenes ^a^
*V. jasicida* 1-TCBS-A	JBIHSE000000000.1	6,106,211	117	5	45.0	5499	5253	12, 12, 11	129	4	78
*V. barjaei* 1-TCBS-B	JBIHSF000000000.1	5,739,451	32	12	44.2	5278	5084	9, 10, 8	108	4	55
*V. alginolyticus* 1-TCBS-C	JBIHSG000000000.1	5,270,144	141	6	44.5	4818	4519	13, 12, 12	129	4	129
*V. alginolyticus* 1-TCBS-D	JBIHSH000000000.1	5,112,826	157	5	44.7	4656	4312	13, 12, 12	129	4	174
*V. alginolyticus* 3-TSA-A	JBIHSI000000000.1	5,168,198	156	5	44.5	4685	4414	10, 9, 9	125	4	114
*V. rumoiensis* 4-MA-B	JBIHSJ000000000.1	3,932,657	88	4	41.9	3578	3378	9, 8, 8	93	4	78
*V. alginolyticus* 4-TSA-C	JBIHSK000000000.1	5,175,393	52	8	44.6	4698	4442	10, 9, 8	118	4	107
*V. diabolicus* 5-MA-A1	JBIHSL000000000.1	5,300,992	155	4	44.7	4913	4632	9, 9, 9	122	5	127
*V. furnissii* 6-MA-B	JBIHSM000000000.1	5,087,888	160	5	50.4	4762	4503	8, 9, 9	109	4	120
*V. alginolyticus* 11-TSA-B2	JBIHSQ000000000.1	5,142,241	155	2	44.6	4685	4405	13, 11, 12	126	4	114
*V. rumoiensis* 14-MA-B	JBIHSN000000000.1	3,992,184	146	5	41.9	3665	3453	9, 8, 8	93	5	89
*V. diabolicus* 15-MA-B	JBIHSR000000000.1	5,149,397	156	2	44.8	4713	4389	11, 12, 12	128	4	157
*V. alginolyticus* 15-TSA-B2	JBIHSS000000000.1	5,117,107	155	3	44.6	4637	4358	12, 11, 12	126	4	114
*V. owensii* 34-PA-B	JBIHSO000000000.1	6,001,683	44	12	45.6	5418	5210	7, 10, 9	124	4	54
*V. alginolyticus* 34-TSA-A	JBIHSP000000000.1	5,186,208	283	3	44.6	4720	4268	13, 12, 12	127	4	284

^a^ the number of pseudogenes indicated includes genes with ambiguous residues, frameshifted genes, incomplete genes, genes with internal stops, or multiple other problems.

**Table 4:** Probabilities of human pathogenicity predicted in the genomes of *Vibrio* strains using the PathogenFinder server (https://cge.food.dtu.dk/services/PathogenFinder/; accessed on 13 October 2024).

	Probability of Being a Human Pathogen	Matched Pathogenic Families	Prediction
*V. jasicida* 1-TCBS-A	0.715	24	Human pathogen
*V. barjaei* 1-TCBS-B	0.457	10	Non-human pathogen
*V. alginolyticus* 1-TCBS-C	0.838	48	Human pathogen
*V. alginolyticus* 1-TCBS-D	0.830	44	Human pathogen
*V. alginolyticus* 3-TSA-A	0.825	40	Human pathogen
*V. rumoiensis* 4-MA-B	0.417	4	Non-human pathogen
*V. alginolyticus* 4-TSA-C	0.849	44	Human pathogen
*V. diabolicus* 5-MA-A1	0.861	49	Human pathogen
*V. furnissii* 6-MA-B	0.756	37	Human pathogen
*V. alginolyticus* 11-TSA-B2	0.849	50	Human pathogen
*V. rumoiensis* 14-MA-B	0.535	8	Human pathogen
*V. diabolicus* 15-MA-B	0.864	49	Human pathogen
*V. alginolyticus* 15-TSA-B2	0.839	44	Human pathogen
*V. owensii* 34-PA-B	0.706	28	Human pathogen
*V. alginolyticus* 34-TSA-A	0.828	42	Human pathogen

## Data Availability

All the data available are included in the manuscript.

## Supplementary Materials

The following supporting information can be downloaded at https://www.mdpi.com/article/10.3390/pathogens14010052/s1, Table S1: Prophages detected in the genome of the *Vibrio* strains, Table S2: Antimicrobial resistance (AMR) genes detected in the *Vibrio* genomes using the Comprehensive Antibiotic Resistance Database (https://card.mcmaster.ca/analyze/rgi (accessed on 13 October 2024)), Table S3: Virulence factor-related genes predicted in the *Vibrio* genomes using the Virulence Factor Database (VFDB) (http://www.mgc.ac.cn/cgi-bin/VFs/v5/main.cgi (accessed on 13 October 2024)), Table S4: Matched pathogenic families in the genomes of *Vibrio* strains in PathogenFinder server (https://cge.food.dtu.dk/services/PathogenFinder/ (accessed on 13 October 2024)), Table S5: CRISPR-associated protein (Cas)-coding genes with CRISPR sequences detected on the CRISPRCasFinder (https://crisprcas.i2bc.paris-saclay.fr/CrisprCasFinder/Index (accessed on 13 October 2024)) server, Table S6: Genomic islands detected by the IslandCompare web tool (https://islandcompare.ca/ (accessed on 13 October 2024)).
