# Supplementary material for: Genomic Insight into Vibrio Isolates from Fresh Raw Mussels and Ready-to-Eat Stuffed Mussels
Source: Pathogens. 2025 Jan 10;14(1):52. doi: 10.3390/pathogens14010052 (PMC11768812; doi:10.3390/pathogens14010052)
Supplement: Supplementary file 1 [file pathogens-14-00052-s001.zip › Table S1.pdf]

Table S1. Prophages detected in the genome of the *Vibrio* strains.

Strain	Phage Region	Region Length (Kb)	Completeness	Total Proteins	Phage Similarity
***V. jasicida* 1-TCBS-A**	1	9,3	intact	15	PHAGE_Vibrio_VfO3K6_NC_002362 (7)
***V. jasicida* 1-TCBS-A**	2	19,7	intact	23	PHAGE_Aeromo_phiO18P_NC_009542 (9)
***V. barjaei* 1-TCBS-B**	1	18,3	intact	19	PHAGE_Vibrio_vB_VpaM_MAR_NC_019722 (5)
***V. alginolyticus* 1-TCBS-C**	1	49,9	questionable	54	PHAGE_Escher_RCS47_NC_042128 (7)
***V. alginolyticus* 1-TCBS-C**	2	12,2	questionable	17	PHAGE_Entero_P1_NC_005856 (9)
***V. alginolyticus* 1-TCBS-C**	3	12,2	incomplete	15	PHAGE_Salmon_SJ46_NC_031129 (7)
***V. alginolyticus* 1-TCBS-D**	1	55,6	intact	52	PHAGE_Entero_SfV_NC_003444 (8)
***V. alginolyticus* 3-TSA-A**	1	40	intact	32	PHAGE_Entero_DE3_NC_042057 (7)
***V. alginolyticus* 3-TSA-A**	2	9,5	intact	17	PHAGE_Vibrio_VCY_phi_NC_016162 (10)
***V. rumoiensis* 4-MA-B**	1	5,4	questionable	15	PHAGE_Entero_lambda_NC_001416 (6)
***V. alginolyticus* 4-TSA-C**	1	11,4	intact	13	PHAGE_Vibrio_VFJ_NC_021562 (9)
***V. alginolyticus* 4-TSA-C**	2	10,5	incomplete	18	PHAGE_Entero_P1_NC_005856 (4)
***V. diabolicus* 5-MA-A1**	1	8,3	questionable	10	PHAGE_Vibrio_VGJphi_NC_004736 (5)
***V. furnissii* 6-MA-B**	1	50,2	intact	71	PHAGE_Pseudo_PMG1_NC_016765 (9)
***V. furnissii* 6-MA-B**	2	41,7	intact	50	PHAGE_Vibrio_Valm_yong1_NC_049477 (26)
***V. alginolyticus* 11-TSA-B2**	1	38,1	intact	22	PHAGE_Entero_HK225_NC_019717 (4)
***V. rumoiensis* 14-MA-B**	1	63,6	intact	54	PHAGE_Halomo_phiHAP_1_NC_010342 (10)
***V. diabolicus* 15-MA-B**	1	60,2	intact	45	PHAGE_Vibrio_K139_NC_003313 (25)
***V. alginolyticus* 15-TSA-B2**	1	34,2	incomplete	27	PHAGE_Escher_500465_1_NC_049342 (7)
***V. owensii* 34-PA-B**	1	43	intact	38	PHAGE_Vibrio_vB_VpaM_MAR_NC_019722 (9)
***V. alginolyticus* 34-TSA-A**	1	29,3	intact	30	PHAGE_Entero_DE3_NC_042057 (6)
***V. alginolyticus* 34-TSA-A**	2	9,5	intact	17	PHAGE_Vibrio_VCY_phi_NC_016162 (10)

The prophage regions detected are assigned a completeness score based on the proportion of phage genes in the identified region, i.e., intact (score > 90); questionable (score 70-90); incomplete (score < 70).
