# Supplementary material for: Genomic Insight into Vibrio Isolates from Fresh Raw Mussels and Ready-to-Eat Stuffed Mussels
Source: Pathogens. 2025 Jan 10;14(1):52. doi: 10.3390/pathogens14010052 (PMC11768812; doi:10.3390/pathogens14010052)
Supplement: Supplementary file 1 [file pathogens-14-00052-s001.zip › Table S2.pdf]

Table S2. Antimicrobial resistance (AMR) genes detected in the *Vibrio* genomes using the Comprehensive Antibiotic Resistance Database (https://card.mcmaster.ca/analyze/rgi).

Type	Rgi criteria	Aro term	Snp	Detection criteria	AMR gene family	Drug class	Resistance mechanism	Identity of matching region (%)	Length of reference sequence (%)
***V. jasicida* 1-TCBS-A**	Strict	*CRP*		Protein homolog model	Resistance-nodulation-cell division (RND) antibiotic efflux pump	Macrolide, fluoroquinolone, penam	Antibiotic efflux	95.24	100.00
	Strict	*Escherichia coli parE* conferring resistance to fluoroquinolones	D476N	Protein variant model	Fluoroquinolone resistant *parE*	Fluoroquinolone	Antibiotic target alteration	78.98	99.37
***V. barjaei* 1-TCBS-B**	Strict	*CRP*		Protein homolog model	Resistance-nodulation-cell division (RND) antibiotic efflux pump	Macrolide, fluoroquinolone, penam	Antibiotic efflux	94.76	100.00
	Strict	*Escherichia coli parE* conferring resistance to fluoroquinolones	D476N	Protein variant model	Fluoroquinolone resistant *parE*	Fluoroquinolone	Antibiotic target alteration	78.34	99.37
***V. alginolyticus* 1-TCBS-C**	Strict	*TxR*		Protein homolog model	ATP-binding cassette (ABC) antibiotic efflux pump	Tetracycline	Antibiotic efflux	85.48	99.69
	Strict	*adeF*		Protein homolog model	Resistance-nodulation-cell division (RND) antibiotic efflux pump	Fluoroquinolone, tetracycline	Antibiotic efflux	61.23	98.77

	Strict	*FosG*		Protein homolog model	Fosfomycin thiol transferase	Phosphonic acid	Antibiotic inactivation	62.12	100.00
	Strict	*CARB-42*		Protein homolog model	CARB beta-lactamase	Penam	Antibiotic inactivation	99.65	100.00
	Strict	*CRP*		Protein homolog model	Resistance-nodulation-cell division (RND) antibiotic efflux pump	Macrolide, fluoroquinolone, penam	Antibiotic efflux	95.24	100.00
	Strict	*Escherichia coli parE* conferring resistance to fluoroquinolones	D476N	Protein variant model	Fluoroquinolone resistant *parE*	Fluoroquinolone	Antibiotic target alteration	78.66	99.37
***V. alginolyticus* 1-TCBS-D**	Perfect	*CARB-42*		Protein homolog model	CARB beta-lactamase	Penam	Antibiotic inactivation	100.0	100.00
	Strict	*adeF*		Protein homolog model	Resistance-nodulation-cell division (RND) antibiotic efflux pump	Fluoroquinolone, tetracycline	Antibiotic efflux	61.23	98.77
	Strict	*CRP*		Protein homolog model	Resistance-nodulation-cell division (RND) antibiotic efflux pump	Macrolide, fluoroquinolone, penam	Antibiotic efflux	95.24	100.00
	Strict	*Escherichia coli parE* conferring resistance to fluoroquinolones	D476N	Protein variant model	Fluoroquinolone resistant *parE*	Fluoroquinolone	Antibiotic target alteration	78.66	99.37

	Strict	*Escherichia coli parE* conferring resistance to fluoroquinolones	D476N	Protein variant model	Fluoroquinolone resistant *parE*	Fluoroquinolone	Antibiotic target alteration	78.47	138.10
***V. alginolyticus* 3-TSA-A**	Perfect	*CARB-42*		Protein homolog model	CARB beta-lactamase	Penam	Antibiotic inactivation	100.0	100.00
	Strict	*adeF*		Protein homolog model	Resistance-nodulation-cell division (RND) antibiotic efflux pump	Fluoroquinolone, tetracycline	Antibiotic efflux	61.23	98.77
	Strict	*CRP*		Protein homolog model	Resistance-nodulation-cell division (RND) antibiotic efflux pump	Macrolide, fluoroquinolone, penam	Antibiotic efflux	95.24	100.00
	Strict	*Escherichia coli parE* conferring resistance to fluoroquinolones	D476N	Protein variant model	Fluoroquinolone resistant *parE*	Fluoroquinolone	Antibiotic target alteration	78.66	99.37
***V. rumoiensis* 4-MA-B**	Strict	*CRP*		Protein homolog model	Resistance-nodulation-cell division (RND) antibiotic efflux pump	Macrolide, fluoroquinolone, penam	Antibiotic efflux	94.29	100.00
	Strict	*QnrC*		Protein homolog model	Quinolone resistance protein (*qnr*)	Fluoroquinolone	Antibiotic target protection	93.64	100.00
***V. alginolyticus* 4-TSA-C**	Perfect	*CARB-42*		Protein homolog model	CARB beta-lactamase	Penam	Antibiotic inactivation	100.0	100.00

	Strict	*adeF*		Protein homolog model	Resistance-nodulation-cell division (RND) antibiotic efflux pump	Fluoroquinolone, tetracycline	Antibiotic efflux	61.14	98.77
	Strict	*CRP*		Protein homolog model	Resistance-nodulation-cell division (RND) antibiotic efflux pump	Macrolide, fluoroquinolone, penam	Antibiotic efflux	95.24	100.00
	Strict	*Escherichia coli parE* conferring resistance to fluoroquinolones	D476N	Protein variant model	Fluoroquinolone resistant *parE*	Fluoroquinolone	Antibiotic target alteration	78.66	99.37
***V. diabolicus* 5-MA-A1**	Strict	*CARB-56*		Protein homolog model	CARB beta-lactamase	Penam	Antibiotic inactivation	96.34	96.47
	Strict	*FosG*		Protein homolog model	Fosfomycin thiol transferase	Phosphonic acid	Antibiotic inactivation	59.85	100.00
	Strict	*TxR*		Protein homolog model	ATP-binding cassette (ABC) antibiotic efflux pump	Tetracycline	Antibiotic efflux	86.29	99.69
	Strict	*CRP*		Protein homolog model	Resistance-nodulation-cell division (RND) antibiotic efflux pump	Macrolide, fluoroquinolone, penam	Antibiotic efflux	95.24	100.00
	Strict	*Escherichia coli parE* conferring	D476N	Protein variant model	Fluoroquinolone resistant *parE*	Fluoroquinolone	Antibiotic target alteration	78.5	99.37

		resistance to fluoroquinolones							
***V. furnissii* 6-MA-B**	Strict	*TxR*		Protein homolog model	ATP-binding cassette (ABC) antibiotic efflux pump	Tetracycline	Antibiotic efflux	66.22	109.43
	Strict	*CRP*		Protein homolog model	Resistance-nodulation-cell division (RND) antibiotic efflux pump	Macrolide, fluoroquinolone, penam	Antibiotic efflux	95.24	100.00
	Strict	*Escherichia coli parE* conferring resistance to fluoroquinolones	D476N	Protein variant model	Fluoroquinolone resistant *parE*	Fluoroquinolone	Antibiotic target alteration	79.62	99.37
***V. alginolyticus* 11-TSA-B2**	Perfect	*CARB-42*		Protein homolog model	CARB beta-lactamase	Penam	Antibiotic inactivation	100.0	100.00
	Strict	*CRP*		Protein homolog model	Resistance-nodulation-cell division (RND) antibiotic efflux pump	Macrolide, fluoroquinolone, penam	Antibiotic efflux	95.24	100.00
	Strict	*adeF*		Protein homolog model	Resistance-nodulation-cell division (RND) antibiotic efflux pump	Fluoroquinolone, tetracycline	Antibiotic efflux	61.15	97.36
	Strict	*Escherichia coli parE* conferring resistance to fluoroquinolones	D476N	Protein variant model	Fluoroquinolone resistant *parE*	Fluoroquinolone	Antibiotic target alteration	78.66	99.37

***V. rumoiensis* 14-MA-B**	Strict	*CRP*	Protein homolog model	Resistance-nodulation-cell division (RND) antibiotic efflux pump	Macrolide, fluoroquinolone, penam	Antibiotic efflux	94.29	100.00
	Strict	*QnrC*	Protein homolog model	Quinolone resistance protein (*qnr*)	Fluoroquinolone	Antibiotic target protection	93.64	100.00
	Strict	*TxR*	Protein homolog model	ATP-binding cassette (ABC) antibiotic efflux pump	Tetracycline	Antibiotic efflux	86.29	99.69
***V. diabolicus* 15-MA-B**	Strict	*TxR*	Protein homolog model	ATP-binding cassette (ABC) antibiotic efflux pump	Tetracycline	Antibiotic efflux	86.29	99.69
	Strict	*CRP*	Protein homolog model	Resistance-nodulation-cell division (RND) antibiotic efflux pump	Macrolide, fluoroquinolone, penam	Antibiotic efflux	95.24	100.00
	Strict	*CARB-56*	Protein homolog model	CARB beta-lactamase	Penam	Antibiotic inactivation	96.11	100.00
***V. alginolyticus* 15-TSA-B2**	Strict	*adeF*	Protein homolog model	Resistance-nodulation-cell division (RND) antibiotic efflux pump	Fluoroquinolone, tetracycline	Antibiotic efflux	61.35	97.36
	Strict	*CRP*	Protein homolog model	Resistance-nodulation-cell division (RND)	Macrolide, fluoroquinolone, penam	Antibiotic efflux	95.24	100.00

					antibiotic efflux pump				
	Strict	*TxR*		Protein homolog model	ATP-binding cassette (ABC) antibiotic efflux pump	Tetracycline	Antibiotic efflux	85.48	99.69
	Strict	*qacG*		Protein homolog model	Small multidrug resistance (SMR) antibiotic efflux pump	Disinfecting agents & antiseptics	Antibiotic efflux	34.95	110.28
	Strict	*CARB-42*		Protein homolog model	CARB beta-lactamase	Penam	Antibiotic inactivation	99.65	100.00
	Strict	*Escherichia coli parE* conferring resistance to fluoroquinolones	D476N	Protein variant model	Fluoroquinolone resistant *parE*	Fluoroquinolone	Antibiotic target alteration	78.66	99.37
***V. owensii* 34-PA-B**	Strict	*adeF*		Protein homolog model	Resistance-nodulation-cell division (RND) antibiotic efflux pump	Fluoroquinolone, tetracycline	Antibiotic efflux	61.62	98.58
	Strict	*CRP*		Protein homolog model	Resistance-nodulation-cell division (RND) antibiotic efflux pump	Macrolide, fluoroquinolone, penam	Antibiotic efflux	95.24	100.00
	Strict	*Escherichia coli parE* conferring resistance to fluoroquinolones	D476N	Protein variant model	Fluoroquinolone resistant *parE*	Fluoroquinolone	Antibiotic target alteration	78.98	99.37

***V. alginolyticus* 34-TSA-A**	Perfect	*CARB-42*	Protein homolog model	CARB beta-lactamase	Penam	Antibiotic inactivation	100.0	100.00
	Strict	*CRP*	Protein homolog model	Resistance-nodulation-cell division (RND) antibiotic efflux pump	Macrolide, fluoroquinolone, penam	Antibiotic efflux	95.24	100.00
