# Supplementary material for: Genomic Insight into Vibrio Isolates from Fresh Raw Mussels and Ready-to-Eat Stuffed Mussels
Source: Pathogens. 2025 Jan 10;14(1):52. doi: 10.3390/pathogens14010052 (PMC11768812; doi:10.3390/pathogens14010052)
Supplement: Supplementary file 1 [file pathogens-14-00052-s001.zip › Table S4.pdf]

Table S4. Matched pathogenic families in the genomes of *Vibrio* strains in PathogenFinder server (https://cge.food.dtu.dk/services/PathogenFinder/).

Strain Name	Protein	Gene	Regions / Sites	Functions
***V. jasicida* 1-TCBS-A**	conserved hypothetical protein	*VPA0001*	HTH_55 / DUF3346	RctB helix turn helix domain / Protein of unknown function (DUF3346)
	putative thiamine ABC transporter, permease protein	*VP0317*	thiP	thiamine transporter membrane protein
	putative chitoporin	*VP0760*	gram_neg_porins	Porins form aqueous channels for the diffusion of small hydrophillic molecules across the outer membrane. Individual 16-strand anti-parallel beta-barrels form a central pore, and trimerizes thru mainly hydrophobic interactions at the interface. / trimer interface [polypeptide binding] / eyelet of channel [active]
	hypothetical protein	*VP2624*	Trm11	tRNA G10 N-methylase Trm11 [Translation, ribosomal structure and biogenesis]
	transcriptional regulator, LysR family	*VP3063*	PRK11074	putative DNA-binding transcriptional regulator
	putative amino acid ABC transporter, permease protein	*VP3066*	HisM	ABC-type amino acid transport system, permease component [Amino acid transport and metabolism]
	conserved hypothetical protein	*VPA0308*	NUDIX_NadM_like	bifunctional NMN adenylyltransferase /ADP-ribose pyrophosphatase and similar proteins
	hypothetical protein	*VPA0193*	DUF2913	Protein of unknown function (DUF2913)
	putative outer membrane protein	*VP0944*	OMP_b-brl	Outer membrane protein beta-barrel domain
	hypothetical protein	*VPA1636*	HTH_XRE	Helix-turn-helix XRE-family like proteins / sequence-specific DNA binding site [nucleotide binding]
	hypothetical protein	*VP2584*	EbsC	Cys-tRNA(Pro) deacylase, prolyl-tRNA editing enzyme YbaK/EbsC [Translation, ribosomal structure and biogenesis]
	conserved hypothetical protein	*VPA0143*	PRK11020	YibL family ribosome-associated protein

	hypothetical protein	*VP1264*	SulA	Cell division inhibitor SulA, prevents FtsZ ring assembly [Cell cycle control, cell division, chromosome partitioning]
	bacteriophage f237 ORF2	*VP1552*	DUF1293	Protein of unknown function (DUF1293)
	RelB protein	*VP1842*	DUF1778	Protein of unknown function (DUF1778)
	hypothetical protein	*VP0026*	ND	ND
***V. barjaei* 1-TCBS-B**	putative helicase	ND	HepA / Mrr_cat	Superfamily II DNA or RNA helicase, SNF2 family / Restriction endonuclease
	methionyl-tRNA synthetase	*metG*	metG	methionyl-tRNA synthetase
	conserved hypothetical protein	ND	EH_Signature	EH_Signature domain
***V. alginolyticus* 1-TCBS-C**	collagenase	*VPA0459*	Peptidase_M9_N / Peptidase_M9 / PPC / PPC	Peptidase family M9 N-terminal / Collagenase / Bacterial pre-peptidase C-terminal domain / Bacterial pre-peptidase C-terminal domain
	conserved hypothetical protein	*VPA0001*	HTH_55 / DUF3346	RctB helix turn helix domain / Protein of unknown function (DUF3346)
	conserved hypothetical protein	VP2376	YejM	Periplasmic protein PbgA/YejM, regulator of the LPS biosynthesis, AlkP superfamily [Cell wall/membrane/envelope biogenesis, Signal transduction mechanisms]
	peptide ABC transporter, periplasmic peptide-binding protein"	*VP1171*	PBP2_DppA_like	The substrate-binding component of an ABC-type dipeptide import system contains the type 2 periplasmic binding fold / peptide binding site [polypeptide binding]
	putative thiamine ABC transporter, permease *protein*	*VP0317*	thiP	thiamine transporter membrane protein

	putative translocator protein PopB	*VP1657*	SseC	Type III secretion system effector SseC (host membrane pore formation) [Intracellular trafficking, secretion, and vesicular transport]
	hypothetical protein	ND	PIN_12	PIN domain
	putative chitoporin	*VP0760*	gram_neg_porins	Porins form aqueous channels for the diffusion of small hydrophillic molecules across the outer membrane. Individual 16-strand anti-parallel beta-barrels form a central pore, and trimerizes thru mainly hydrophobic interactions at the interface. / trimer interface [polypeptide binding] / eyelet of channel [active]
	hypothetical protein	*VP2624*	Trm11	tRNA G10 N-methylase Trm11 [Translation, ribosomal structure and biogenesis]
	transcriptional regulator, LacI family	*VP2632*	PurR	DNA-binding transcriptional regulator, LacI/PurR family [Transcription]
	hypothetical protein SCH_145 (plasmid)	ND	AbiH	Bacteriophage abortive infection AbiH
	transcriptional regulator, LysR family	*VP3063*	PRK11074	putative DNA-binding transcriptional regulator
	hypothetical protein	*VPA0211*	ND	ND
	hypothetical protein	*VPA0064*	MBL_fold_Vmh	Vmh family MBL fold metallo-hydrolase
	hypothetical protein	*VP1284*	HTH_XRE	Helix-turn-helix XRE-family like proteins. Prokaryotic DNA binding proteins belonging to the xenobiotic response element family of transcriptional regulators / non-specific DNA binding site [nucleotide binding] / salt bridge [active] / sequence-specific DNA binding site [nucleotide binding]
	putative amino acid ABC transporter, permease protein	*VP3066*	HisM	ABC-type amino acid transport system, permease component [Amino acid transport and metabolism]
	putative transcriptional regulator	*VPA0593*	IclR	DNA-binding transcriptional regulator, IclR family [Transcription]

	conserved hypothetical protein	*VPA0308*	NUDIX_NadM_like	bifunctional NMN adenylyltransferase/ADP-ribose pyrophosphatase and similar proteins
	accessory colonization factor AcfA	*VPA0695*	AcfA_fam_omp	AcfA family outer membrane beta-barrel protein
	hypothetical protein	*VP0658*	PilW	Type IV pilus assembly protein PilW [Cell motility, Extracellular structures]
	hypothetical protein	*VPA0093*	Haloacid Dehalogenase-like Hydrolases	The haloacid dehalogenase (HAD) superfamily includes carbon and phosphorus hydrolases such as 2-haloalkonoate dehalogenase, epoxide hydrolase, phosphoserine phosphatase, phosphomannomutase, phosphoglycolate phosphatase, P-type ATPase, among others
	hypothetical protein	*VPA0193*	DUF2913	Protein of unknown function (DUF2913)
	hypothetical protein	*VPA0779*	COG3911	Predicted ATPase [General function prediction only]
	putative hypothetical protein	*VPA1737*	MscS	Small-conductance mechanosensitive channel [Cell wall/membrane/envelope biogenesis]
	putative outer membrane protein	*VP0944*	OMP_b-brl	Outer membrane protein beta-barrel domain
	hypothetical protein	*VP1066*	YgiB	Uncharacterized conserved protein YgiB, UPF0441/DUF1190 family, involved in bioifilm formation [Function unknown]
	hypothetical protein	*VP2667*	LptC_YrbK	LPS export ABC transporter periplasmic protein LptC
	hypothetical protein	*VPA0383*	cupin_DAD	2,4'-Dihydroxyacetophenone dioxygenase (DAD), cupin domain / tetramer interface [polypeptide binding] / putative active site [active]
	putative outer membrane lipoprotein Pcp	*VP1192*	SlyB	Outer membrane lipoprotein SlyB [Cell wall/membrane/envelope biogenesis]
	conserved hypothetical protein	*VPA0143*	PRK11020	YibL family ribosome-associated protein
	hypothetical protein	*VP1264*	SulA	Cell division inhibitor SulA, prevents FtsZ ring assembly [Cell cycle control, cell division, chromosome partitioning]

	mazG-related protein	*VPA0776*	MazG	MazG nucleotide pyrophosphohydrolase domain
	LafC	*VPA1551*	FliS / coiled coil / coiled coil / coiled coil / coiled coil	Flagellin-specific chaperone FliS [Cell motility, Intracellular trafficking, secretion, and vesicular transport] / heterodimer interface [polypeptide binding] / coiled coil [structural motif] / coiled coil [structural motif] / coiled coil [structural motif] / coiled coil [structural motif]
	hypothetical protein	*VP1625*	ND	ND
	hypothetical protein	*VP2837*	VanZ	VanZ-like family protein (function unknown)
	hypothetical protein	*VPA0279*	DUF3012	Protein of unknown function (DUF3012)
	hypothetical protein	*VP1977*	DUF4250	Domain of unknown function (DUF4250)
	hypothetical protein	*VPA0676*	ND	ND
	hypothetical protein	*VP1922*	ND	ND
	Trp operon leader peptide	*VP1955*	Trp_leader2	Tryptophan operon leader peptide
***V. alginolyticus* 1-TCBS-D**	collagenase	*VPA0459*	Peptidase_M9_N / Peptidase_M9 / PPC / PPC	Peptidase family M9 N-terminal / Collagenase / Bacterial pre-peptidase C-terminal domain / Bacterial pre-peptidase C-terminal domain
	conserved hypothetical protein	*VPA0001*	HTH_55 / DUF3346	RctB helix turn helix domain / Protein of unknown function (DUF3346)
	conserved hypothetical protein	*VP2376*	YejM	Periplasmic protein PbgA/YejM, regulator of the LPS biosynthesis, AlkP superfamily [Cell wall/membrane/envelope biogenesis, Signal transduction mechanisms]
	peptide ABC transporter, periplasmic peptide-binding protein	*VP1171*	PBP2_DppA_like	The substrate-binding component of an ABC-type dipeptide import system contains the type 2 periplasmic binding fold / peptide binding site [polypeptide binding]
	putative thiamine ABC transporter, permease protein	*VP0317*	thiP	thiamine transporter membrane protein

	putative translocator protein PopB	*VP1657*	SseC	Type III secretion system effector SseC (host membrane pore formation) [Intracellular trafficking, secretion, and vesicular transport]
	putative chitoporin	*VP0760*	gram_neg_porins /	Porins form aqueous channels for the diffusion of small hydrophillic molecules across the outer membrane. Individual 16-strand anti-parallel beta-barrels form a central pore, and trimerizes thru mainly hydrophobic interactions at the interface. / trimer interface [polypeptide binding] / eyelet of channel [active]
	hypothetical protein	*VP2624*	Trm11	tRNA G10 N-methylase Trm11 [Translation, ribosomal structure and biogenesis]
	transcriptional regulator, LacI family	*VP2632*	PurR	DNA-binding transcriptional regulator, LacI/PurR family [Transcription]
	transcriptional regulator, LysR family	*VP3063*	PRK11074	putative DNA-binding transcriptional regulator
	hypothetical protein	*VPA0064*	MBL_fold_Vmh	Vmh family MBL fold metallo-hydrolase
	hypothetical protein	*VP1284*	HTH_XRE /	Helix-turn-helix XRE-family like proteins. Prokaryotic DNA binding proteins belonging to the xenobiotic response element family of transcriptional regulators / non-specific DNA binding site [nucleotide binding] / salt bridge [active] / sequence-specific DNA binding site [nucleotide binding]
	conserved hypothetical protein	*VPA1140*	YHI9	Predicted epimerase YddE/YHI9, PhzF superfamily [General function prediction only]
	putative amino acid ABC transporter, permease protein	*VP3066*	HisM	ABC-type amino acid transport system, permease component [Amino acid transport and metabolism]
	putative transcriptional regulator	*VPA0593*	IclR	DNA-binding transcriptional regulator, IclR family [Transcription]
	conserved hypothetical protein	*VPA0308*	NUDIX_NadM_like	bifunctional NMN adenylyltransferase/ADP-ribose pyrophosphatase and similar proteins

	accessory colonization factor AcfA	*VPA0695*	AcfA_fam_omp	AcfA family outer membrane beta-barrel protein
	hypothetical protein	*VPA0093*	Haloacid Dehalogenase-like Hydrolases	The haloacid dehalogenase (HAD) superfamily includes carbon and phosphorus hydrolases such as 2-haloalkonoate dehalogenase, epoxide hydrolase, phosphoserine phosphatase, phosphomannomutase, phosphoglycolate phosphatase, P-type ATPase, among others.
	hypothetical protein	*VPA0193*	DUF2913	Protein of unknown function (DUF2913)
	hypothetical protein	*VPA0779*	COG3911	Predicted ATPase [General function prediction only]
	putative hypothetical protein	*VPA1737*	MscS	Small-conductance mechanosensitive channel [Cell wall/membrane/envelope biogenesis]
	putative outer membrane protein	*VP0944*	OMP_b-brl	Outer membrane protein beta-barrel domain
	hypothetical protein	*VP1066*	YgiB	Uncharacterized conserved protein YgiB, UPF0441/DUF1190 family, involved in bioifilm formation [Function unknown]
	hypothetical protein	*VP2667*	LptC_YrbK	LPS export ABC transporter periplasmic protein LptC
	hypothetical protein	*VP1430*	ND	ND
	hypothetical protein	*VPA0383*	cupin_DAD / other	2,4'-Dihydroxyacetophenone dioxygenase (DAD), cupin domain / tetramer interface [polypeptide binding] / putative active site [active]
	putative outer membrane lipoprotein Pcp	*VP1192*	SlyB	Outer membrane lipoprotein SlyB [Cell wall/membrane/envelope biogenesis]
	conserved hypothetical protein	*VPA0143*	PRK11020	YibL family ribosome-associated protein
	hypothetical protein	*VP1264*	SulA	Cell division inhibitor SulA, prevents FtsZ ring assembly [Cell cycle control, cell division, chromosome partitioning]
	mazG-related protein	*VPA0776*	MazG	MazG nucleotide pyrophosphohydrolase domain

	LafC	*VPA1551*	FliS / other / coiled coil / coiled coil / coiled coil / coiled coil	Flagellin-specific chaperone FliS [Cell motility,Intracellular trafficking, secretion, and vesicular transport] / heterodimer interface [polypeptide binding] / coiled coil [structural motif] / coiled coil [structural motif] / coiled coil [structural motif] / coiled coil [structural motif]
	hypothetical protein	*VP1625*	ND	ND
	hypothetical protein	*VP2837*	VanZ	VanZ-like family protein (function unknown)
	RelB protein	*VP1842*	DUF1778	Protein of unknown function (DUF1778)
	hypothetical protein	*VPA0279*	DUF3012	Protein of unknown function (DUF3012)
	hypothetical protein	*VP1977*	DUF4250	Domain of unknown function (DUF4250)
	hypothetical protein	*VPA0676*	ND	ND
	hypothetical protein	*VP1922*	ND	ND
	Trp operon leader peptide	*VP1955*	Trp_leader2	Tryptophan operon leader peptide
***V. alginolyticus* 3-TSA-A**	collagenase	*VPA0459*	Peptidase_M9_N / Peptidase_M9 / PPC / PPC	Peptidase family M9 N-terminal / Collagenase / Bacterial pre-peptidase C-terminal domain / Bacterial pre-peptidase C-terminal domain
	conserved hypothetical protein	*VPA0001*	HTH_55 / DUF3346	RctB helix turn helix domain / Protein of unknown function (DUF3346)
	conserved hypothetical protein	*VP2376*	YejM	Periplasmic protein PbgA/YejM, regulator of the LPS biosynthesis, AlkP superfamily [Cell wall/membrane/envelope biogenesis, Signal transduction mechanisms]
	peptide ABC transporter, periplasmic peptide-binding protein	*VP1171*	PBP2_DppA_like	The substrate-binding component of an ABC-type dipeptide import system contains the type 2 periplasmic binding fold / peptide binding site [polypeptide binding]
	putative thiamine ABC transporter, permease protein	*VP0317*	thiP	thiamine transporter membrane protein

	putative translocator protein PopB	*VP1657*	SseC	Type III secretion system effector SseC (host membrane pore formation) [Intracellular trafficking, secretion, and vesicular transport]
	hypothetical protein	*VP2624*	Trm11	tRNA G10 N-methylase Trm11 [Translation, ribosomal structure and biogenesis]
	transcriptional regulator, LacI family	*VP2632*	PurR	DNA-binding transcriptional regulator, LacI/PurR family [Transcription]
	transcriptional regulator, LysR family	*VP3063*	PRK11074	putative DNA-binding transcriptional regulator
	hypothetical protein	*VPA0064*	MBL_fold_Vmh	Vmh family MBL fold metallo-hydrolase
	hypothetical protein	*VP1284*	HTH_XRE	Helix-turn-helix XRE-family like proteins. Prokaryotic DNA binding proteins belonging to the xenobiotic response element family of transcriptional regulators / non-specific DNA binding site [nucleotide binding] / salt bridge [active] / sequence-specific DNA binding site [nucleotide binding]
	putative transcriptional regulator	*VPA0593*	IclR	DNA-binding transcriptional regulator, IclR family [Transcription]
	conserved hypothetical protein	*VPA0308*	NUDIX_NadM_like	bifunctional NMN adenylyltransferase/ADP-ribose pyrophosphatase and similar proteins
	accessory colonization factor AcfA	*VPA0695*	AcfA_fam_omp	AcfA family outer membrane beta-barrel protein
	hypothetical protein	*VPA0093*	Haloacid Dehalogenase-like Hydrolases	The haloacid dehalogenase (HAD) superfamily includes carbon and phosphorus hydrolases such as 2-haloalkonoate dehalogenase, epoxide hydrolase, phosphoserine phosphatase, phosphomannomutase, phosphoglycolate phosphatase, P-type ATPase, among others
	putative hypothetical protein	*VPA1737*	MscS	Small-conductance mechanosensitive channel [Cell wall/membrane/envelope biogenesis]
	putative outer membrane protein	*VP0944*	OMP_b-brl	Outer membrane protein beta-barrel domain
	hypothetical protein	*VP2667*	LptC_YrbK	LPS export ABC transporter periplasmic protein LptC

	hypothetical protein	*VP1430*	ND	ND
	hypothetical protein	*VPA1636*	HTH_XRE	Helix-turn-helix XRE-family like proteins / equence-specific DNA binding site [nucleotide binding]
	hypothetical protein	*VPA0383*	cupin_DAD	2,4'-Dihydroxyacetophenone dioxygenase (DAD), cupin domain / tetramer interface [polypeptide binding] / putative active site [active]
	putative outer membrane lipoprotein Pcp	*VP1192*	SlyB	Outer membrane lipoprotein SlyB [Cell wall/membrane/envelope biogenesis]
	conserved hypothetical protein	*VPA0143*	PRK11020	YibL family ribosome-associated protein
	hypothetical protein	*VP1264*	SulA	Cell division inhibitor SulA, prevents FtsZ ring assembly [Cell cycle control, cell division, chromosome partitioning]
	mazG-related protein	*VPA0776*	MazG	MazG nucleotide pyrophosphohydrolase domain
	LafC	*VPA1551*	FliS / coiled coil / coiled coil / coiled coil / coiled coil	Flagellin-specific chaperone FliS [Cell motility Intracellular trafficking, secretion, and vesicular transport] / heterodimer interface [polypeptide binding] / coiled coil [structural motif]
	hypothetical protein	*VP1625*	ND	ND
	hypothetical protein	*VP2837*	VanZ	VanZ-like family protein (function unknown)
	RelB protein	*VP1842*	DUF1778	Protein of unknown function (DUF1778)
	hypothetical protein	*VPA0279*	DUF3012	Protein of unknown function (DUF3012)
	putative transcription regulator protein	*VP2144*	HTH_XRE	Helix-turn-helix XRE-family like proteins. Prokaryotic DNA binding proteins belonging to the xenobiotic response element family of transcriptional regulators / non-specific DNA binding site [nucleotide binding] / salt bridge [active] / sequence-specific DNA binding site [nucleotide binding]
	hypothetical protein	*VP1977*	DUF4250	Domain of unknown function (DUF4250)
	hypothetical protein	*VPA0676*	ND	ND
	hypothetical protein	*VP1922*	ND	ND

	Trp operon leader peptide	*VP1955*	Trp_leader2	Tryptophan operon leader peptide
***V. rumoiensis* 4-MA-B**	hypothetical protein	ND	ND	ND
	hypothetical protein	ND	ND	ND
***V. alginolyticus* 4-TSA-C**	collagenase	*VPA0459*	Peptidase_M9_N / Peptidase_M9 / PPC / PPC	Peptidase family M9 N-terminal / Collagenase / Bacterial pre-peptidase C-terminal domain / Bacterial pre-peptidase C-terminal domain
	conserved hypothetical protein	*VPA0001*	HTH_55 / DUF3346	RctB helix turn helix domain; pfam18622 / Protein of unknown function (DUF3346)
	conserved hypothetical protein	*VP2376*	YejM	Periplasmic protein PbgA/YejM, regulator of the LPS biosynthesis, AlkP superfamily [Cell wall/membrane/envelope biogenesis, Signal transduction mechanisms]
	putative thiamine ABC transporter, permease protein	*VP0317*	thiP	thiamine transporter membrane protein
	putative translocator protein PopB	*VP1657*	SseC	Type III secretion system effector SseC (host membrane pore formation) [Intracellular trafficking, secretion, and vesicular transport]
	putative chitoporin	*VP0760*	gram_neg_porins	Porins form aqueous channels for the diffusion of small hydrophillic molecules across the outer membrane. Individual 16-strand anti-parallel beta-barrels form a central pore, and trimerizes thru mainly hydrophobic interactions at the interface. / trimer interface [polypeptide binding] / eyelet of channel [active]
	hypothetical protein	*VP2624*	Trm11	tRNA G10 N-methylase Trm11 [Translation, ribosomal structure and biogenesis]
	transcriptional regulator, LacI family	*VP2632*	PurR	DNA-binding transcriptional regulator, LacI/PurR family [Transcription]
	transcriptional regulator, LysR family	*VP3063*	PRK11074	putative DNA-binding transcriptional regulator
	hypothetical protein	*VPA0064*	MBL_fold_Vmh	Vmh family MBL fold metallo-hydrolase

	hypothetical protein	*VP1284*	HTH_XRE	Helix-turn-helix XRE-family like proteins. Prokaryotic DNA binding proteins belonging to the xenobiotic response element family of transcriptional regulators / non-specific DNA binding site [nucleotide binding] / salt bridge [active] / sequence-specific DNA binding site [nucleotide binding]
	putative amino acid ABC transporter, permease protein	*VP3066*	HisM	ABC-type amino acid transport system, permease component [Amino acid transport and metabolism]
	putative transcriptional regulator	*VPA0593*	IclR	DNA-binding transcriptional regulator, IclR family [Transcription]
	conserved hypothetical protein	*VPA0308*	NUDIX_NadM_like	bifunctional NMN adenylyltransferase/ADP-ribose pyrophosphatase and similar proteins
	putative resolvase	*VP0638*	SR_ResInv / HTH_23	Serine Recombinase (SR) family, Resolvase and Invertase subfamily, catalytic domain; members contain a C-terminal DNA binding domain. Serine recombinases catalyze site-specific recombination of DNA molecules by a concerted, four-strand cleavage. / catalytic residues [active] / catalytic nucleophile [active] / Presynaptic Site I dimer interface [polypeptide binding] / Synaptic Antiparallel dimer interface [polypeptide binding] / Synaptic Flat tetramer interface [polypeptide binding] / Synaptic Site I dimer interface [polypeptide binding] / DNA binding site [nucleotide binding] / Homeodomain-like domain
	hypothetical protein	*VP0658*	PilW	Type IV pilus assembly protein PilW [Cell motility, Extracellular structures]
	hypothetical protein	*VPA0093*	Haloacid Dehalogenase-like Hydrolases	The haloacid dehalogenase (HAD) superfamily includes carbon and phosphorus hydrolases such as 2-haloalkonoate dehalogenase, epoxide hydrolase, phosphoserine phosphatase, phosphomannomutase, phosphoglycolate phosphatase, P-type ATPase, among others.

	putative fimbrial protein Z, transcriptional regulator (LuxR/UhpA family)	*VPA0741*	PRK09935	fimbriae biosynthesis transcriptional regülatör FimZ
	hypothetical protein	*VP0641*	ND	ND
	hypothetical protein	*VPA0193*	DUF2913	Protein of unknown function (DUF2913)
	putative hypothetical protein	*VPA1737*	MscS	Small-conductance mechanosensitive channel [Cell wall/membrane/envelope biogenesis]
	putative outer membrane protein	*VP0944*	OMP_b-brl	Outer membrane protein beta-barrel domain
	hypothetical protein	*VP2667*	LptC_YrbK	LPS export ABC transporter periplasmic protein LptC
	hypothetical protein	*VPA0383*	cupin_DAD	2,4'-Dihydroxyacetophenone dioxygenase (DAD), cupin domain / tetramer interface [polypeptide binding] / putative active site [active]
	putative ribonuclease HI	*VP0642*	PRK08719	ribonuclease H / RNA/DNA hybrid binding site [nucleotide binding]
	putative outer membrane lipoprotein Pcp	*VP1192*	SlyB	Outer membrane lipoprotein SlyB [Cell wall/membrane/envelope biogenesis]
	conserved hypothetical protein	*VPA0143*	PRK11020	YibL family ribosome-associated protein
	hypothetical protein	*VP1264*	SulA	Cell division inhibitor SulA, prevents FtsZ ring assembly [Cell cycle control, cell division, chromosome partitioning]
	mazG-related protein	*VPA0776*	MazG	MazG nucleotide pyrophosphohydrolase domain
	LafC	*VPA1551*	FliS / coiled coil / coiled coil / coiled coil / coiled coil	Flagellin-specific chaperone FliS [Cell motility Intracellular trafficking, secretion, and vesicular transport] / heterodimer interface [polypeptide binding] / coiled coil [structural motif]
	hypothetical protein	*VP1625*	ND	ND
	hypothetical protein	*VP2837*	VanZ	VanZ-like family protein (function unknown)
	RelB protein	*VP1842*	DUF1778	Protein of unknown function (DUF1778)

	hypothetical protein	*VPA0279*	DUF3012	Protein of unknown function (DUF3012)
	hypothetical protein	*VP1977*	DUF4250	Domain of unknown function (DUF4250)
	hypothetical protein	*VP0639*	ND	ND
	hypothetical protein	*VPA0676*	ND	ND
	hypothetical protein	*VP1922*	ND	ND
	Trp operon leader peptide	*VP1955*	Trp_leader2	Tryptophan operon leader peptide
***V. diabolicus* 5-MA-A1**	collagenase	*VPA0459*	Peptidase_M9_N / Peptidase_M9 / PPC / PPC	Peptidase family M9 N-terminal / Collagenase / Bacterial pre-peptidase C-terminal domain / Bacterial pre-peptidase C-terminal domain
	putative 83 kDa decaheme outer membrane cytochrome c	*VP1220*	decahem_SO1788	decaheme c-type cytochrome, OmcA/MtrC family /
	heme transport protein HutA	*VPA0882*	OM_channels	Porin superfamily. These outer membrane channels share a beta-barrel structure that differ in strand and shear number. Classical (gram-negative) porins are non-specific channels for small hydrophillic molecules and form 16 beta-stranded barrels / N-terminal plug [active]
	putative outer membrane protein precursor	*VP1218*	OMP_MtrB_PioB	decaheme-associated outer membrane protein, MtrB/PioB family
	conserved hypothetical protein	*VPA0001*	HTH_55 / DUF3346	RctB helix turn helix domain / Protein of unknown function (DUF3346)
	conserved hypothetical protein	*VP2376*	YejM	Periplasmic protein PbgA/YejM, regulator of the LPS biosynthesis, AlkP superfamily [Cell wall/membrane/envelope biogenesis, Signal transduction mechanisms]
	putative transporter binding protein	*VPA0957*	SgrR_N / PBP2_NikA_DppA_OppA_like	Sugar transport-related sRNA regulator N-term / The substrate-binding domain of an ABC-type nickel/oligopeptide-like import system contains the type 2 periplasmic binding fold

	putative thiamine ABC transporter, permease protein	*VP0317*	thiP	thiamine transporter membrane protein
	putative chitinase A	*VPA1177*	ChiA	hitinase, GH18 family [Carbohydrate transport and metabolism]
	putative translocator protein PopB	*VP1657*	SseC	Type III secretion system effector SseC (host membrane pore formation) [Intracellular trafficking, secretion, and vesicular transport]
	hypothetical protein	*VP1567*	ND	ND
	putative chitoporin	*VP0760*	gram_neg_porins	Porins form aqueous channels for the diffusion of small hydrophillic molecules across the outer membrane. Individual 16-strand anti-parallel beta-barrels form a central pore, and trimerizes thru mainly hydrophobic interactions at the interface. / trimer interface [polypeptide binding] / eyelet of channel [active]
	hypothetical protein	*VP2624*	Trm11	tRNA G10 N-methylase Trm11 [Translation, ribosomal structure and biogenesis]
	transcriptional regulator, LacI family	*VP2632*	PurR	DNA-binding transcriptional regulator, LacI/PurR family [Transcription]
	transcriptional regulator, LysR family	*VP3063*	PRK11074	putative DNA-binding transcriptional regulator
	hypothetical protein	*VPA0064*	MBL_fold_Vmh	Vmh family MBL fold metallo-hydrolase
	hypothetical protein	*VP1284*	HTH_XRE, salt bridge [active],	Helix-turn-helix XRE-family like proteins. Prokaryotic DNA binding proteins belonging to the xenobiotic response element family of transcriptional regulators / non-specific DNA binding site [nucleotide binding] / salt bridge [active] / sequence-specific DNA binding site [nucleotide binding]
	putative amino acid ABC transporter, permease protein	*VP3066*	HisM	ABC-type amino acid transport system, permease component [Amino acid transport and metabolism]

	putative transcriptional regulator	*VPA0593*	IclR	DNA-binding transcriptional regulator, IclR family [Transcription]
	conserved hypothetical protein	*VPA0308*	NUDIX_NadM_like	bifunctional NMN adenylyltransferase/ADP-ribose pyrophosphatase and similar proteins
	putative cytochrome subunit of sulfide dehydrogenase precursor	*VP1221*	CytC553	Cytochrome c553 [Energy production and conversion]
	hypothetical protein	*VPA0093*	Haloacid Dehalogenase-like Hydrolases	The haloacid dehalogenase (HAD) superfamily includes carbon and phosphorus hydrolases such as 2-haloalkonoate dehalogenase, epoxide hydrolase, phosphoserine phosphatase, phosphomannomutase, phosphoglycolate phosphatase, P-type ATPase, among others
	hypothetical protein	*VPA0779*	COG3911	Predicted ATPase [General function prediction only]
	putative hypothetical protein	*VPA1737*	MscS	Small-conductance mechanosensitive channel [Cell wall/membrane/envelope biogenesis]
	putative outer membrane protein	*VP0944*	OMP_b-brl	Outer membrane protein beta-barrel domain
	hypothetical protein	*VP1066*	YgiB	Uncharacterized conserved protein YgiB, UPF0441/DUF1190 family, involved in bioifilm formation [Function unknown]
	hypothetical protein	*VP2667*	LptC_YrbK	LPS export ABC transporter periplasmic protein LptC
	hypothetical protein	*VPA1636*	HTH_XRE	Helix-turn-helix XRE-family like proteins / sequence-specific DNA binding site [nucleotide binding]
	hypothetical protein	*VPA0383*	cupin_DAD	2,4'-Dihydroxyacetophenone dioxygenase (DAD), cupin domain / tetramer interface[polypeptide binding] / putative active site [active]
	hypothetical protein	*VPA1570*	DUF2799 / PilN	Protein of unknown function (DUF2799) / Type IV pilus assembly protein PilN [Cell motility, Extracellular structures]

	putative MSHA pilin protein MshC	*VP2696*	PulG	Type II secretory pathway, pseudopilin PulG [Cell motility, Intracellular trafficking, secretion, and vesicular transport, Extracellular structures]
	putative structural protein P5 (Alteromonas phage PM2)	*VP1566*	V	virion protein; Provisional
	hypothetical protein	*VPA0391*	GloA	Catechol 2,3-dioxygenase or related enzyme, vicinal oxygen chelate (VOC) family [Secondary metabolites biosynthesis, transport and catabolism]
	conserved hypothetical protein	*VPA0143*	PRK11020	YibL family ribosome-associated protein
	hypothetical protein	*VP1264*	SulA	Cell division inhibitor SulA, prevents FtsZ ring assembly [Cell cycle control, cell division, chromosome partitioning]
	mazG-related protein	*VPA0776*	MazG	MazG nucleotide pyrophosphohydrolase domain
	LafC	*VPA1551*	FliS / coiled coil / coiled coil / coiled coil / coiled coil	Flagellin-specific chaperone FliS [Cell motility, Intracellular trafficking, secretion, and vesicular transport] / heterodimer interface [polypeptide binding] / coiled coil [structural motif] / coiled coil [structural motif] / coiled coil [structural motif] / coiled coil [structural motif] / coiled coil [structural motif]
	hypothetical protein	*VP2837*	VanZ	VanZ-like family protein (function unknown)
	hypothetical protein	*VP1574*	ND	ND
	hypothetical protein	*VPA0279*	DUF3012	Protein of unknown function (DUF3012)
	hypothetical protein	*VPA0998*	ND	ND
	hypothetical protein	*VP1977*	DUF4250	Domain of unknown function (DUF4250)
	hypothetical protein	*VPA1081*	ND	ND
	hypothetical protein	*VPA0676*	ND	ND
***V. furnissii* 6-MA-B**	IncF plasmid conjugative transfer protein TraG	ND	TraG_N	TraG-like protein, N-terminal region

	hypothetical protein VCD_003681	ND	WGR	WGR domain
	putative transcriptional regulator	ND	IclR	biotype: Ogawa / DNA-binding transcriptional regulator, IclR family [Transcription]
	hypothetical protein VCD_003674	ND	ND	ND
	hypothetical protein VCD_001100	ND	ND	ND
	hypothetical protein VCD_003687	ND	ND	ND
	hypothetical protein VCD_003749	ND	ND	ND
***V. alginolyticus* 11-TSA-B2**	collagenase	*VPA0459*	Peptidase_M9_N / Peptidase_M9 / PPC / PPC	Peptidase family M9 N-terminal / Collagenase / Bacterial pre-peptidase C-terminal domain / Bacterial pre-peptidase C-terminal domain
	conserved hypothetical protein	*VPA0001*	HTH_55 / DUF3346	RctB helix turn helix domain / Protein of unknown function (DUF3346)
	conserved hypothetical protein	*VP2376*	YejM	Periplasmic protein PbgA/YejM, regulator of the LPS biosynthesis, AlkP superfamily [Cell wall/membrane/envelope biogenesis, Signal transduction mechanisms]
	putative replication initiation protein, phage-related	*VPA0908*	Phage_GPA	Bacteriophage replication gene A protein (GPA)
	putative thiamine ABC transporter, permease protein	*VP0317*	thiP	thiamine transporter membrane protein
	putative translocator protein PopB	*VP1657*	SseC	Type III secretion system effector SseC (host membrane pore formation) [Intracellular trafficking, secretion, and vesicular transport]
	putative chitoporin	*VP0760*	gram_neg_porins /	Porins form aqueous channels for the diffusion of small hydrophillic molecules across the outer membrane. Individual 16-strand anti-parallel beta-

				barrels form a central pore, and trimerizes thru mainly hydrophobic interactions at the interface.
	hypothetical protein	*VP2624*	Trm11	tRNA G10 N-methylase Trm11 [Translation, ribosomal structure and biogenesis]
	transcriptional regulator, LacI family	*VP2632*	PurR	DNA-binding transcriptional regulator, LacI/PurR family [Transcription]
	transcriptional regulator, LysR family	*VP3063*	PRK11074	putative DNA-binding transcriptional regulator
	hypothetical protein	*VPA0211*	ND	ND
	hypothetical protein	*VPA0064*	MBL_fold_Vmh	Vmh family MBL fold metallo-hydrolase
	putative transmembrane protein	*VP0400*	Mrr / YrdD	Restriction endonuclease Mrr [Defense mechanisms]/ DNA topoisomerase I, ssDNA-binding Zn-finger and Zn-ribbon domains [Replication, recombination and repair]
	hypothetical protein	*VP1284*	HTH_XRE /	Helix-turn-helix XRE-family like proteins. Prokaryotic DNA binding proteins belonging to the xenobiotic response element family of transcriptional regulators / non-specific DNA binding site [nucleotide binding] / sequence-specific DNA binding site [nucleotide binding] /
	putative amino acid ABC transporter, permease protein	*VP3066*	HisM	BC-type amino acid transport system, permease component [Amino acid transport and metabolism]
	putative transcriptional regulator	*VPA0593*	IclR	DNA-binding transcriptional regulator, IclR family [Transcription]
	conserved hypothetical protein	*VPA0308*	NUDIX_NadM_like	bifunctional NMN adenylyltransferase/ADP-ribose pyrophosphatase and similar proteins
	hypothetical protein	*VPA0093*	Haloacid Dehalogenase-like Hydrolases	The haloacid dehalogenase (HAD) superfamily includes carbon and phosphorus hydrolases such as 2-haloalkonoate dehalogenase, epoxide hydrolase, phosphoserine phosphatase, phosphomannomutase, phosphoglycolate phosphatase, P-type ATPase, among others

	hypothetical protein	*VPA0193*	DUF2913	Protein of unknown function (DUF2913)
	putative hypothetical protein	*VPA1737*	MscS	Small-conductance mechanosensitive channel [Cell wall/membrane/envelope biogenesis]
	putative outer membrane protein	*VP0944*	OMP_b-brl	Outer membrane protein beta-barrel domain
	hypothetical protein	*VP1066*	YgiB	Uncharacterized conserved protein YgiB,UPF0441/DUF1190 family, involved in bioifilm formation [Function unknown]
	hypothetical protein	*VP2667*	LptC_YrbK	LPS export ABC transporter periplasmic protein LptC
	hypothetical protein	*VP1430*	ND	ND
	hypothetical protein	*VPA1636*	HTH_XRE	Helix-turn-helix XRE-family like proteins / sequence-specific DNA binding site [nucleotide binding]
	hypothetical protein	*VPA0383*	cupin_DAD	2,4'-Dihydroxyacetophenone dioxygenase (DAD), cupin domain / tetramer interface [polypeptide binding] / putative active site [active]
	putative outer membrane lipoprotein Pcp	*VP1192*	SlyB	Outer membrane lipoprotein SlyB [Cell wall/membrane/envelope biogenesis]
	putative structural protein P5 (Alteromonas phage PM2)	*VP1566*	V	virion protein; Provisional
	conserved hypothetical protein	*VPA0143*	PRK11020	YibL family ribosome-associated protein
	hypothetical protein	*VP1264*	SulA	Cell division inhibitor SulA, prevents FtsZ ring assembly [Cell cycle control, cell division, chromosome partitioning]
	mazG-related protein	*VPA0776*	MazG	MazG nucleotide pyrophosphohydrolase domain
	LafC	*VPA1551*	FliS / coiled coil / coiled coil / coiled coil / coiled coil	Flagellin-specific chaperone FliS [Cell motility,Intracellular trafficking, secretion, and vesicular transport] / heterodimer interface [polypeptide binding] / coiled coil [structural motif] / coiled coil [structural motif] / coiled coil [structural

				motif] / coiled coil [structural motif] / coiled coil [structural motif]
	hypothetical protein	*VP1625*	ND	ND
	hypothetical protein	*VP2837*	VanZ	VanZ-like family protein (function unknown)
	RelB protein	*VP1842*	DUF1778	Protein of unknown function (DUF1778)
	hypothetical protein	*VP1574*	ND	ND
	hypothetical protein	*VP1584*	ND	ND
	hypothetical protein	*VPA0279*	DUF3012	Protein of unknown function (DUF3012)
	putative transcription regulator protein	*VP2144*	HTH_XRE	Helix-turn-helix XRE-family like proteins. Prokaryotic DNA binding proteins belonging to the xenobiotic response element family of transcriptional regulators / non-specific DNA binding site [nucleotide binding]/ sequence-specific DNA binding site [nucleotide binding]
	hypothetical protein	*VP1977*	DUF4250	Domain of unknown function (DUF4250)
	hypothetical protein	*VPA0676*	ND	ND
	hypothetical protein	*VP1922*	ND	ND
	Trp operon leader peptide	*VP1955*	Trp_leader2	Tryptophan operon leader peptide
***V. rumoiensis* 14-MA-B**	methionyl-tRNA synthetas	*metG*	metG	ND
	hypothetical protein	ND	Inovirus_Gp2	ND
	hypothetical protein	ND	ND	ND
	hypothetical protein	ND	ND	ND
***V. diabolicus* 15-MA-B**	collagenase	*VPA0459*	Peptidase_M9_N / Peptidase_M9 / PPC / PPC	Peptidase family M9 N-terminal / Collagenase / Bacterial pre-peptidase C-terminal domain / Bacterial pre-peptidase C-terminal domain
	putative 83 kDa decaheme outer membrane cytochrome c	*VP1220*	decahem_SO1788	decaheme c-type cytochrome, OmcA/MtrC family
	heme transport protein HutA	*VPA0882*	OM_channels	Porin superfamily. These outer membrane channels share a beta-barrel structure that differ in strand and shear number. Classical (gram-negative) porins are

				non-specific channels for small hydrophillic molecules and form 16 beta-stranded barrels
	putative outer membrane protein precursor	*VP1218*	OMP_MtrB_PioB	decaheme-associated outer membrane protein, MtrB/PioB family
	conserved hypothetical protein	*VPA0001*	HTH_55 / DUF3346	RctB helix turn helix domain / Protein of unknown function (DUF3346)
	conserved hypothetical protein	*VP2376*	YejM	Periplasmic protein PbgA/YejM, regulator of the LPS biosynthesis, AlkP superfamily [Cell wall/membrane/envelope biogenesis, Signal transduction mechanisms]
	putative transporter binding protein	*VPA0957*	SgrR_N / PBP2_NikA_DppA_OppA_like	Sugar transport-related sRNA regulator N-term / The substrate-binding domain of an ABC-type nickel/oligopeptide-like import system contains the type 2 periplasmic binding fold
	putative thiamine ABC transporter, permease protein	*VP0317*	thiP	thiamine transporter membrane protein
	putative regulatory protein UhpC	*VPA0824*	MFS_OPA_SLC37	Organophosphate: Pi antiporter/Solute Carrier family 37 of the Major Facilitator Superfamily of transporters / putative chemical substrate binding pocket [chemical binding]
	putative chitinase A	*VPA1177*	ChiA	Chitinase, GH18 family [Carbohydrate transport and metabolism]
	putative translocator protein PopB	*VP1657*	SseC	Type III secretion system effector SseC (host membrane pore formation) [Intracellular trafficking, secretion, and vesicular transport]
	putative chitoporin	*VP0760*	gram_neg_porins	Porins form aqueous channels for the diffusion of small hydrophillic molecules across the outer membrane. Individual 16-strand anti-parallel beta-barrels form a central pore, and trimerizes thru mainly hydrophobic interactions at the interface. / trimer

				interface [polypeptide binding] / eyelet of channel [active]
	hypothetical protein	*VP2624*	Trm11	tRNA G10 N-methylase Trm11 [Translation, ribosomal structure and biogenesis]
	transcriptional regulator, LacI family	*VP2632*	PurR	DNA-binding transcriptional regulator, LacI/PurR family [Transcription]
	transcriptional regulator, LysR family	*VP3063*	PRK11074	putative DNA-binding transcriptional regulator
	hypothetical protein	*VPA0064*	MBL_fold_Vmh	Vmh family MBL fold metallo-hydrolase
	lateral flagellin LafA	*VPA1548*	lat_flg_LafA_2	lateral flagellin LafA
	hypothetical protein	*VP1284*	HTH_XRE	Helix-turn-helix XRE-family like proteins. Prokaryotic DNA binding proteins belonging to the xenobiotic response element family of transcriptional regulators / non-specific DNA binding site [nucleotide binding] / salt bridge [active] / sequence-specific DNA binding site [nucleotide binding]
	putative amino acid ABC transporter, permease protein	*VP3066*	HisM	ABC-type amino acid transport system, permease component [Amino acid transport and metabolism]
	putative transcriptional regulator	*VPA0593*	IclR	DNA-binding transcriptional regulator, IclR family [Transcription]
	conserved hypothetical protein	*VPA0308*	NUDIX_NadM_like	bifunctional NMN adenylyltransferase/ADP-ribose pyrophosphatase and similar proteins
	putative resolvase	*VP0638*	SR_ResInv /	Serine Recombinase (SR) family, Resolvase and Invertase subfamily, catalytic domain; members contain a C-terminal DNA binding domain. Serine recombinases catalyze site-specific recombination of DNA molecules by a concerted, four-strand cleavage / catalytic residues / catalytic nucleophile [active] / Presynaptic Site I dimer interface [polypeptide binding] / Synaptic Antiparallel dimer interface [polypeptide binding] / Synaptic Flat tetramer interface [polypeptide binding] / Synaptic Site I dimer

				interface [polypeptide binding] / DNA binding / Homeodomain-like domain
	putative cytochrome subunit of sulfide dehydrogenase precursor	*VP1221*	CytC553	Cytochrome c553 [Energy production and conversion]
	hypothetical protein	*VPA0093*	Haloacid Dehalogenase-like Hydrolases	The haloacid dehalogenase (HAD) superfamily includes carbon and phosphorus hydrolases such as 2-haloalkonoate dehalogenase, epoxide hydrolase, phosphoserine phosphatase, phosphomannomutase, phosphoglycolate phosphatase, P-type ATPase, among others.
	hypothetical protein	*VP0641*	ND	ND
	putative hypothetical protein	*VPA1737*	MscS	Small-conductance mechanosensitive channel [Cell wall/membrane/envelope biogenesis]
	putative outer membrane protein	*VP0944*	OMP_b-brl	Outer membrane protein beta-barrel domain
	hypothetical protein	*VP1066*	YgiB	Uncharacterized conserved protein YgiB, UPF0441/DUF1190 family, involved in bioifilm formation [Function unknown]
	hypothetical protein	*VP2667*	LptC_YrbK	LPS export ABC transporter periplasmic protein LptC
	hypothetical protein	*VPA1636*	HTH_XRE	Helix-turn-helix XRE-family like proteins / sequence-specific DNA binding site [nucleotide binding]
	hypothetical protein	*VPA0383*	cupin_DAD	2,4'-Dihydroxyacetophenone dioxygenase (DAD), cupin domain / tetramer interface [polypeptide binding] / putative active site [active]
	hypothetical protein	*VPA1570*	DUF2799 / PilN	Protein of unknown function (DUF2799)/ Type IV pilus assembly protein PilN [Cell motility, Extracellular structures]
	putative ribonuclease HI	*VP0642*	PRK08719	ribonuclease H / RNA/DNA hybrid binding site [nucleotide binding]
	conserved hypothetical protein	*VPA0143*	PRK11020	YibL family ribosome-associated protein

	mazG-related protein	*VPA0776*	MazG	MazG nucleotide pyrophosphohydrolase domain
	LafC	*VPA1551*	FliS / coiled coil / coiled coil / coiled coil / coiled coil /	Flagellin-specific chaperone FliS [Cell motility, Intracellular trafficking, secretion, and vesicular transport] / heterodimer interface [polypeptide binding] / coiled coil [structural motif] / / coiled coil [structural motif] / coiled coil [structural motif] / coiled coil [structural motif] /
	hypothetical protein	*VP2837*	VanZ	VanZ-like family protein (function unknown)
	hypothetical protein	*VPA0279*	DUF3012	Protein of unknown function (DUF3012)
	hypothetical protein	*VPA0998*	ND	ND
	hypothetical protein	*DUF4250*	ND	ND
	hypothetical protein	*VP0639*	ND	ND
	hypothetical protein	*VPA0676*	ND	ND
	hypothetical protein	*VP1740*	ND	ND
	Trp operon leader peptide	*VP1955*	Trp_leader2	Tryptophan operon leader peptide
***V. alginolyticus* 15-TSA-B2**	collagenase	*VPA0459*	Peptidase_M9_N / Peptidase_M9 / PPC / PPC	Peptidase family M9 N-terminal / Collagenase / Bacterial pre-peptidase C-terminal domain / Bacterial pre-peptidase C-terminal domain
	conserved hypothetical protein	*VPA0001*	HTH_55 / DUF3346	RctB helix turn helix domain / Protein of unknown function (DUF3346)
	conserved hypothetical protein	*VP2376*	YejM	Periplasmic protein PbgA/YejM, regulator of the LPS biosynthesis, AlkP superfamily [Cell wall/membrane/envelope biogenesis, Signal transduction mechanisms
	peptide ABC transporter, periplasmic peptide-binding protein	*VP1171*	PBP2_DppA_like	The substrate-binding component of an ABC-type dipeptide import system contains the type 2 periplasmic binding fold / peptide binding site [polypeptide binding]
	putative thiamine ABC transporter, permease protein	*VP0317*	thiP	thiamine transporter membrane protein

	putative translocator protein PopB	*VP1657*	SseC	Type III secretion system effector SseC (host membrane pore formation) [Intracellular trafficking, secretion, and vesicular transport]
	putative chitoporin	*VP0760*	gram_neg_porins	Porins form aqueous channels for the diffusion of small hydrophillic molecules across the outer membrane. Individual 16-strand anti-parallel beta-barrels form a central pore, and trimerizes thru mainly hydrophobic interactions at the interface / trimer interface [polypeptide binding]/ eyelet of channel [active]
	hypothetical protein	*VP2624*	Trm11	tRNA G10 N-methylase Trm11 [Translation, ribosomal structure and biogenesis]
	transcriptional regulator, LacI family	*VP2632*	PurR	DNA-binding transcriptional regulator, LacI/PurR family [Transcription]
	transcriptional regulator, LysR family	*VP3063*	PRK11074	putative DNA-binding transcriptional regulator
	hypothetical protein	*VPA0064*	MBL_fold_Vmh	Vmh family MBL fold metallo-hydrolase
	hypothetical protein	*VP1284*	HTH_XRE	Helix-turn-helix XRE-family like proteins. Prokaryotic DNA binding proteins belonging to the xenobiotic response element family of transcriptional regulators / non-specific DNA binding site [nucleotide binding] / sequence-specific DNA binding site [nucleotide binding]
	putative amino acid ABC transporter, permease protein	*VP3066*	HisM	ABC-type amino acid transport system, permease component [Amino acid transport and metabolism]
	putative transcriptional regulator	*VPA0593*	IclR	DNA-binding transcriptional regulator, IclR family [Transcription]
	conserved hypothetical protein	*VPA0308*	NUDIX_NadM_like	bifunctional NMN adenylyltransferase/ADP-ribose pyrophosphatase and similar proteins
	accessory colonization factor AcfA	*VPA0695*	AcfA_fam_omp	AcfA family outer membrane beta-barrel protein

	hypothetical protein	*VP0658*	PilW	Type IV pilus assembly protein PilW [Cell motility, Extracellular structures]
	hypothetical protein	*VPA0093*	Haloacid Dehalogenase-like Hydrolases	The haloacid dehalogenase (HAD) superfamily includes carbon and phosphorus hydrolases such as 2-haloalkonoate dehalogenase, epoxide hydrolase, phosphoserine phosphatase, phosphomannomutase, phosphoglycolate phosphatase, P-type ATPase, among others
	putative hypothetical protein	*VPA1737*	MscS	Small-conductance mechanosensitive channel [Cell wall/membrane/envelope biogenesis]
	putative outer membrane protein	*VP0944*	OMP_b-brl	Outer membrane protein beta-barrel domain
	hypothetical protein	*VP1066*	YgiB	Uncharacterized conserved protein YgiB, UPF0441/DUF1190 family, involved in bioifilm formation [Function unknown]
	hypothetical protein	*VP2667*	LptC_YrbK	LPS export ABC transporter periplasmic protein LptC
	hypothetical protein	*VP1430*	ND	ND
	hypothetical protein	*VPA1636*	HTH_XRE	Helix-turn-helix XRE-family like proteins / equence-specific DNA binding site [nucleotide binding]
	hypothetical protein	*VPA0383*	cupin_DAD /	2,4'-Dihydroxyacetophenone dioxygenase (DAD), cupin Domain / tetramer interface [polypeptide binding] / putative active site [active]
	putative outer membrane lipoprotein Pcp	*VP1192*	SlyB	Outer membrane lipoprotein SlyB [Cell wall/membrane/envelope biogenesis]
	conserved hypothetical protein	*VPA0143*	PRK11020	YibL family ribosome-associated protein
	hypothetical protein	*VP1264*	SulA	Cell division inhibitor SulA, prevents FtsZ ring assembly [Cell cycle control, cell division, chromosome partitioning]
	mazG-related protein	*VPA0776*	MazG	MazG nucleotide pyrophosphohydrolase domain
	LafC	VPA1551	FliS / coiled coil	heterodimer interface [polypeptide binding] / coiled coil [structural motif] / coiled coil [structural motif] /

				coiled coil [structural motif] / coiled coil [structural motif]
	hypothetical protein	*VP1625*	ND	ND
	hypothetical protein	*VP2837*	VanZ	VanZ-like family protein (function unknown)
	hypothetical protein	*VPA0279*	DUF3012	Protein of unknown function (DUF3012)
	hypothetical protein	*VP1977*	DUF4250	Domain of unknown function (DUF4250)
	hypothetical protein	*VPA0676*	ND	ND
	hypothetical protein	*VP1922*	ND	ND
	Trp operon leader peptide	*VP1955*	Trp_leader2	Tryptophan operon leader peptide
***V. owensii* 34-PA-B**	conserved hypothetical protein	*VPA0001*	HTH_55 / DUF3346	RctB helix turn helix domain / Protein of unknown function (DUF3346)
	putative thiamine ABC transporter, permease protein	*VP0317*	thiP	thiamine transporter membrane protein
	putative chitoporin	*VP0760*	gram_neg_porins	Porins form aqueous channels for the diffusion of small hydrophillic molecules across the outer membrane. Individual 16-strand anti-parallel beta-barrels form a central pore, and trimerizes thru mainly hydrophobic interactions at the interface
	putative chitoporin	*VP0760*	gram_neg_porins	trimer interface [polypeptide binding] / eyelet of channel [active]
	hypothetical protein	*VP2624*	Trm11	tRNA G10 N-methylase Trm11 [Translation, ribosomal structure and biogenesis]
	transcriptional regulator, LacI family	*VP2632*	PurR	DNA-binding transcriptional regulator, LacI/PurR family [Transcription]
	hypothetical protein	*VP1284*	HTH_XRE	Helix-turn-helix XRE-family like proteins. Prokaryotic DNA binding proteins belonging to the xenobiotic response element family of transcriptional regulators / non-specific DNA binding site [nucleotide binding]/ equence-specific DNA binding site [nucleotide binding]

	putative amino acid ABC transporter, permease protein	*VP3066*	HisM	ABC-type amino acid transport system, permease component [Amino acid transport and metabolism]
	putative transcriptional regulator	*VPA0593*	IclR	DNA-binding transcriptional regulator, IclR family [Transcription]
	conserved hypothetical protein	*VPA0308*	NUDIX_NadM_like	bifunctional NMN adenylyltransferase/ADP-ribose pyrophosphatase and similar proteins
	hypothetical protein	*VPA0193*	DUF2913	Protein of unknown function (DUF2913)
	putative outer membrane protein	*VP0944*	OMP_b-brl	Outer membrane protein beta-barrel domain
	hypothetical protein	*VP2667*	LptC_YrbK	LPS export ABC transporter periplasmic protein LptC
	hypothetical protein	*VPA1636*	HTH_XRE	Helix-turn-helix XRE-family like proteins / sequence-specific DNA binding site [nucleotide binding]
	hypothetical protein	*VP2584*	EbsC	Cys-tRNA(Pro) deacylase, prolyl-tRNA editing enzyme YbaK/EbsC [Translation, ribosomal structure and biogenesis]
	putative MSHA pilin protein MshC	*VP2696*	PulG	Type II secretory pathway, pseudopilin PulG [Cell motility, Intracellular trafficking, secretion, and vesicular transport, Extracellular structures]
	conserved hypothetical protein	*VPA0143*	PRK11020	YibL family ribosome-associated protein
	hypothetical protein	*VP1264*	SulA	Cell division inhibitor SulA, prevents FtsZ ring assembly [Cell cycle control, cell division, chromosome partitioning]
	NADH dehydrogenase subunit II-related protein	*VP0024*	ND	ND
	hypothetical protein	*VP0026*	ND	ND
	hypothetical protein	*VP1740*	ND	ND
***V. alginolyticus* 34-TSA-A**	collagenase	*VPA0459*	ND	ND
	conserved hypothetical protein	*VPA0001*	ND	ND

	conserved hypothetical protein	*VP2376*	ND	Periplasmic protein PbgA/YejM, regulator of the LPS biosynthesis, AlkP superfamily [Cell wall/membrane/envelope biogenesis, Signal transduction mechanisms]
	peptide ABC transporter, periplasmic peptide-binding protein	*VP1171*	ND	ND
	putative thiamine ABC transporter, permease protein	*VP0317*	thiP	thiamine transporter membrane protein
	putative translocator protein PopB	*VP1657*	SseC	Type III secretion system effector SseC (host membrane pore formation) [Intracellular trafficking, secretion, and vesicular transport]
	putative chitoporin	*VP0760*	gram_neg_porins	trimer interface / eyelet of channel [active]
	hypothetical protein	*VP2624*	Trm11	tRNA G10 N-methylase Trm11 [Translation, ribosomal structure and biogenesis]
	transcriptional regulator, LacI family	*VP2632*	PurR	DNA-binding transcriptional regulator, LacI/PurR family [Transcription]
	transcriptional regulator, LysR family	*VP3063*	PRK11074	putative DNA-binding transcriptional regulator
	hypothetical protein	*VPA0064*	MBL_fold_Vmh	Vmh family MBL fold metallo-hydrolase
	hypothetical protein	*VP1284*	HTH_XRE	non-specific DNA binding site / salt bridge [active]/ sequence-specific DNA binding site [nucleotide binding]
	putative amino acid ABC transporter, permease protein	*VP3066*	HisM	ABC-type amino acid transport system, permease component [Amino acid transport and metabolism]
	putative transcriptional regulator	*VPA0593*	IclR	DNA-binding transcriptional regulator, IclR family [Transcription]
	conserved hypothetical protein	*VPA0308*	NUDIX_NadM_like	bifunctional NMN adenylyltransferase/ADP-ribose pyrophosphatase and similar proteins
	accessory colonization factor AcfA	*VPA0695*	AcfA_fam_omp	AcfA family outer membrane beta-barrel protein

	hypothetical protein	*VPA0093*	Haloacid Dehalogenase-like Hydrolases	The haloacid dehalogenase (HAD) superfamily includes carbon and phosphorus hydrolases such as 2-haloalkonoate dehalogenase, epoxide hydrolase, phosphoserine phosphatase, phosphomannomutase, phosphoglycolate phosphatase, P-type ATPase, among others
	putative outer membrane protein	*VP0944*	OMP_b-brl	Outer membrane protein beta-barrel domain
	hypothetical protein	*VP2667*	LptC_YrbK	LPS export ABC transporter periplasmic protein LptC
	hypothetical protein	*VP1430*	ND	ND
	hypothetical protein	*VPA1636*	HTH_XRE	Helix-turn-helix XRE-family like proteins
	hypothetical protein	*VPA0383*	cupin_DAD	2,4'-Dihydroxyacetophenone dioxygenase (DAD), cupin Domain / putative active site [active]
	putative outer membrane lipoprotein Pcp	*VP1192*	SlyB	Outer membrane lipoprotein SlyB [Cell wall/membrane/envelope biogenesis]
	conserved hypothetical protein	*VPA0143*	PRK11020	YibL family ribosome associated protein
	hypothetical protein	*VP1264*	SulA	Cell division inhibitor SulA, prevents FtsZ ring assembly [Cell cycle control, cell division, chromosome partitioning]
	mazG-related protein	*VPA0776*	MazG	MazG nucleotide pyrophosphohydrolase domain
	LafC	*VPA1551*	FliS<br>coiled coil	Flagellin-specific chaperone FliS [Cell motility, Intracellular trafficking, secretion, and vesicular transport] / coiled coil [structural motif]
	hypothetical protein	*VP1625*	ND	ND
	hypothetical protein	*VP2837*	VanZ	VanZ-like family protein (function unknown)
	RelB protein	*VP1842*	DUF1778	Protein of unknown function (DUF1778)
	hypothetical protein	*VPA0279*	DUF3012	Protein of unknown function (DUF3012)
	putative transcription regulator protein	*VP2144*	HTH_XRE	Helix-turn-helix XRE-family like proteins. Prokaryotic DNA binding proteins belonging to the xenobiotic response element family of transcriptional regulators /

				non-specific DNA binding site [nucleotide binding] / salt bridge [active]
	hypothetical protein	*VP1977*	DUF4250	Domain of unknown function (DUF4250)
	hypothetical protein	*VPA0676*	ND	ND
	hypothetical protein	*VP1922*	ND	ND
	Trp operon leader peptide	*VP1955*	Trp_leader2	Tryptophan operon leader peptide

ND: Not defined
