# Supplementary material for: Genomic Insight into Vibrio Isolates from Fresh Raw Mussels and Ready-to-Eat Stuffed Mussels
Source: Pathogens. 2025 Jan 10;14(1):52. doi: 10.3390/pathogens14010052 (PMC11768812; doi:10.3390/pathogens14010052)
Supplement: Supplementary file 1 [file pathogens-14-00052-s001.zip › Table S5.pdf]

Table S5. CRISPR-associated protein (Cas) coding genes with CRISPR sequences detected on the CRISPRCasFinder (https://crisprcas.i2bc.paris-saclay.fr/CrisprCasFinder/Index) server.

Strain	Element	Start	End	Spacer / Gene	Repeat consensus	Evidence Level
***V. rumoiensis* 4-MA-B**	CRISPR	446529	446676	2	CTTACTAGCCACACACCCGATAGCACAC	1
	CRISPR	417405	417672	4	CTTACTAGCCACACACCCGATAGCACAC	3
***V. rumoiensis* 14-MA-B**	CRISPR	1770088	1770295	3	TTTCCTAGCTGCCTATTCGGCAGGTCAC	1
	CRISPR	129078	129174	1	CGAGGTATTTTTGCAAACACGGA	1
***V. diabolicus* 15-MA-B**	CRISPR	145860	145937	1	TTTTGGAACAATAAAGTTTGTACAC	1
	CRISPR	2967221	2967349	1	GTCATTCCGAGGAGCCTAAGCGACATCAGGAATCT	1
***V. owensii* 34-PA-B**	CRISPR	7164	7296	1	GGCGGGTTCCGCGCTGGTTCCCGAGGCGGGGTCC	1
	CRISPR	730186	730964	10	TTTAACCAAGATATAGGCCATTGGGATAC	2
